# Structural analysis of the *Trypanosoma brucei* EIF4E6/EIF4G5 complex reveals details of the interaction between unusual eIF4F subunits

**DOI:** 10.1038/s41598-024-52364-1

**Published:** 2024-01-25

**Authors:** Renato Ferras Penteado, Renata Santana da Silva, Danielle Maria Nascimento Moura, Gustavo Barbosa de Lima, Amaranta Muniz Malvezzi, Tallyta Tâmara da Silva Monteiro, Camila Cavalcanti Xavier, Sophie Vichier-Guerre, Laurence Dugué, Sylvie Pochet, Nilson Ivo Tonin Zanchin, Christian Robson de Souza Reis, Osvaldo Pompílio de Melo Neto, Beatriz Gomes Guimarães

**Affiliations:** 1 Carlos Chagas Institute – Oswaldo Cruz Foundation, Curitiba, PR Brazil; 2 Aggeu Magalhães Institute – Oswaldo Cruz Foundation, Recife, PE Brazil; 3 Epigenetic Chemical Biology, Institut Pasteur, Paris, France

**Keywords:** Structural biology, X-ray crystallography, Translation

## Abstract

Recognition of the mRNA 5′ end is a critical step needed for translation initiation. This step is performed by the cap binding protein eIF4E, which joins the larger eIF4G subunit to form the eIF4F complex. Trypanosomatids have a minimum of five different eIF4F-like complexes formed through specific but not well-defined interactions between four different eIF4E and five eIF4G homologues. The EIF4E6/EIF4G5 complex has been linked with the stage-specific translation of mRNAs encoding the major *Trypanosoma brucei* virulence factors. Here, to better define the molecular basis for the TbEIF4E6/TbEIF4G5 interaction, we describe the identification of the peptide interacting with TbEIF4E6 in the region comprising residues 79–166 of TbEIF4G5. The TbEIF4E6-TbEIF4G5_79-116 complex reconstituted with recombinant proteins is highly stable even in the absence of cap-4. The crystal structure of the complex was subsequently solved, revealing extensive interacting surfaces. Comparative analyses highlight the conservation of the overall structural arrangement of different eIF4E/eIF4G complexes. However, highly different interacting surfaces are formed with distinct binding contacts occurring both in the canonical and noncanonical elements within eIF4G and the respective eIF4E counterpart. These specific pairs of complementary interacting surfaces are likely responsible for the selective association needed for the formation of distinct eIF4F complexes in trypanosomatids.

## Introduction

mRNA recognition and recruitment by the small ribosomal subunit and associated translation initiation factors is the most critical stage needed for translation to start and proceed in all known organisms^1,2^. In eukaryotes, mRNA recognition is mainly mediated by the cap binding protein eIF4E, which is responsible for binding to the capped 5′ end of the mRNA. eIF4E is part of the eIF4F initiation factor, a complex formed through the interaction between eIF4E and the large eIF4G subunit. A third eIF4F subunit in mammals, more loosely associated in yeast, is eIF4A, an RNA helicase that is needed for the removal of secondary structures found along the 5′ end of the mRNA and that might interfere with translation^3–5^.

eIF4E is a strictly eukaryotic protein whose three-dimensional structure has been solved from different organisms, including humans, revealing the conserved architecture of the so-called eIF4E domain. As first described by Marcotrigiano and collaborators^6,7^, a curved-shaped α/β structure forms the cap binding pocket on the concave surface of the protein, while eIF4G and 4E-interacting proteins bind to the convex side of eIF4E. Both binding activities are dependent on conserved tryptophan/aromatic amino acid residues found in most eIF4E homologues. Binding to the cap is mediated by the stacking of two of those residues that localize to the concave side of eIF4E, while binding to eIF4G involves another tryptophan residue (W73 in human eIF4E-1) mapped to its convex side. Within eIF4Gs and within metazoan eIF4E-binding proteins (4E-BPs), binding to eIF4E generally requires a short, conserved motif that fits into the consensus YX_4_LΦ, with X representing any amino acid and φ meaning a hydrophobic residue. This canonical motif is part of a larger bipartite recognition region, which localizes within the N-terminal region of eIF4G and includes a linker segment followed by a noncanonical binding motif^8–10^.

Parasitic protozoans belonging to the family Trypanosomatidae are divergent eukaryotes that are best known for the various human diseases they cause. They are also characterized by distinct molecular processes generally not seen in most eukaryotes, such as the constitutive polycistronic transcription of their protein-coding genes^11,12^. The trypanosomatid mRNA 5′ end is also unique among other eukaryotic mRNAs due to the modified nature of the 7-methyl-GTP cap. While the cap is generally added to the mRNA cotranscriptionally and consists of a methylated GTP molecule added in an inverted orientation to the 5′ end of the nascent transcript, in trypanosomatids, the cap structure is formed by a methylated GTP molecule, as occurs in other eukaryotes, with additional 2′-O-ribose methylations of the first four residues and base methylation on nucleotides 1 and 4^13^. This hypermodified mRNA 5′-end, called cap-4, is synthesized on the 5′ end of a special RNA precursor^14^, the spliced leader precursor. The first 39 nucleotides of this precursor containing cap-4 functions as a special ‘exon’ that is incorporated into the 5′ region of all mRNAs, originally synthesized as long polycistronic precursor transcripts by a mechanism known as the trans-splicing reaction^14^.

Trypanosomatids have recently emerged as relevant models for the study of eIF4Es and related complexes, with six homologues (EIF4E1 to EIF4E6) conserved in different species but generally more divergent in sequence than better characterized eIF4Es from metazoans, plants and yeasts^15,16^. Two of the trypanosomatid eIF4Es, EIF4E1 and EIF4E2, do not bind eIF4Gs but are associated with specific binding partners, 4E1-IP and SLBP2, respectively, with not yet clearly defined but possible regulatory roles^17–19^. The remaining four eIF4Es all bind to different eIF4G homologues (five are conserved in trypanosomatids), forming a minimum of five different eIF4F complexes^15^. EIF4E3 and EIF4E4, with greater similarity in sequence, bind to related eIF4Gs (EIF4G4 and EIF4G3, respectively), with the resulting eIF4F-like complexes found to be active in translation but binding to different mRNA targets and protein partners^20–22^. EIF4E5 is alternatively found to be associated with EIF4G1 or EIF4G2, plus noncharacterized protein partners, but its function is less understood, although a possible role associated with cell motility has been implied^23^. The most divergent eIF4E in trypanosomatids is EIF4E6, found specifically associated with EIF4G5 as well as with a noncharacterized protein having domains with predicted guanylyltransferase and nucleoside triphosphate activities, named G5-IP^24,25^. RNAi knockdown of *T. brucei* EIF4E6 in procyclic forms did not affect growth but weakened flagellar attachment, while EIF4E6 depletion in bloodstream forms was lethal^24,26^. Both EIF4E6 and EIF4G5 were found to enhance the expression of a reporter mRNA in tethering assays in *T. brucei* bloodstream forms^27,28^ and were also found to be specifically associated with VSG-encoding mRNAs^29^. In contrast, *Leishmania* EIF4E6 was proposed to have a translation-repressive role for the parasite promastigote form^25^.

To date, the crystal structures of three trypanosomatid eIF4E homologues have been reported. The structure of *L. major* EIF4E1 in complex with the 4E1-IP interacting peptide was first described, with binding by 4E1-IP seen to prevent EIF4E1 cap-binding activity^30^. More recently, we determined the crystal structure of *T. cruzi* EIF4E5 in complex with mRNA cap-4 and other cap analogs^31^ and of *L. major* EIF4E5 in the apo form^32^. Structural comparisons showed important differences in both the cap binding pocket and the eIF4G interacting surface, providing evidence of distinct binding mechanisms of the trypanosomatid eIF4Es with their partners^31,32^. Here, we focused on the EIF4E6/EIF4G5 complex, aiming to better define the molecular basis for their interaction. We first set out to identify binding motifs through site-directed mutagenesis and protein‒protein interaction assays, thus managing to infer an EIF4E6 binding segment within the EIF4G5 N-terminal region. Having successfully expressed both EIF4E6 and its EIF4G5 binding peptide in *Escherichia coli*, we then reconstituted the complex in vitro and solved its 3D structure by crystallography. Our analysis reveals an extensive interaction surface and the formation of a highly stable complex. The structural data and comparison with the human homologue indicate specific features for the eIF4E and eIF4G subunits that are likely to be responsible for the selective association between the partners to form eIF4F-like complexes.

## Results

### Investigating the EIF4E6 binding motifs within the *T. brucei* EIF4G5

Orthologues to both EIF4E6 and EIF4G5 are found throughout trypanosomatids and more divergent kinetoplastids^33^, highlighting a critical requirement for this complex, which has been maintained since the early origin of these organisms. A direct interaction between *T. brucei* EIF4E6 and its EIF4G5 partner was first indicated through reciprocal experiments carried out using yeast two-hybrid assays^24,28^. However, the relatively low sequence conservation between trypanosomatids eIF4Es and eIF4Gs and orthologues from other eukaryotes, where the eIF4E-eIF4G interaction has been better defined, does not allow proper identification of the residues involved in the *T. brucei* EIF4E6-4G5 interaction.

The three-dimensional structure of *T. brucei* EIF4G5, as predicted by AlphaFold^34,35^, reveals three globular alpha-helical domains, predicted with high confidence (https://alphafold.ebi.ac.uk/entry/Q57VY5), preceded by a nonstructured N-terminal region (Supplementary Fig. 1). In addition to the MIF4G/HEAT1 domain, which is the signature of the 4G initiation factors, two alpha-helical domains are found at the C-terminal portion of EIF4G5, with a similar topology of the HEAT repeat domains found in the C-terminal of human eIF4G1^36^. A sequence alignment of EIF4G5 orthologues from different kinetoplastid species confirms the conservation of the central MIF4G domain, as well as of the two C-terminal helical domains (Supplementary Fig. 2). Greater conservation among the various sequences is observed for MIF4G/HEAT1, and this is particularly noticeable in more divergent organisms, such as *Bodo saltans*. Elements found within the N-terminal regions of the various EIF4G5 orthologues are also conserved, as well as segments found between the last two predicted helical domains, but there is very little or no conservation in the segment connecting MIF4G/HEAT1 to the second helical domain.

**Figure 1:**
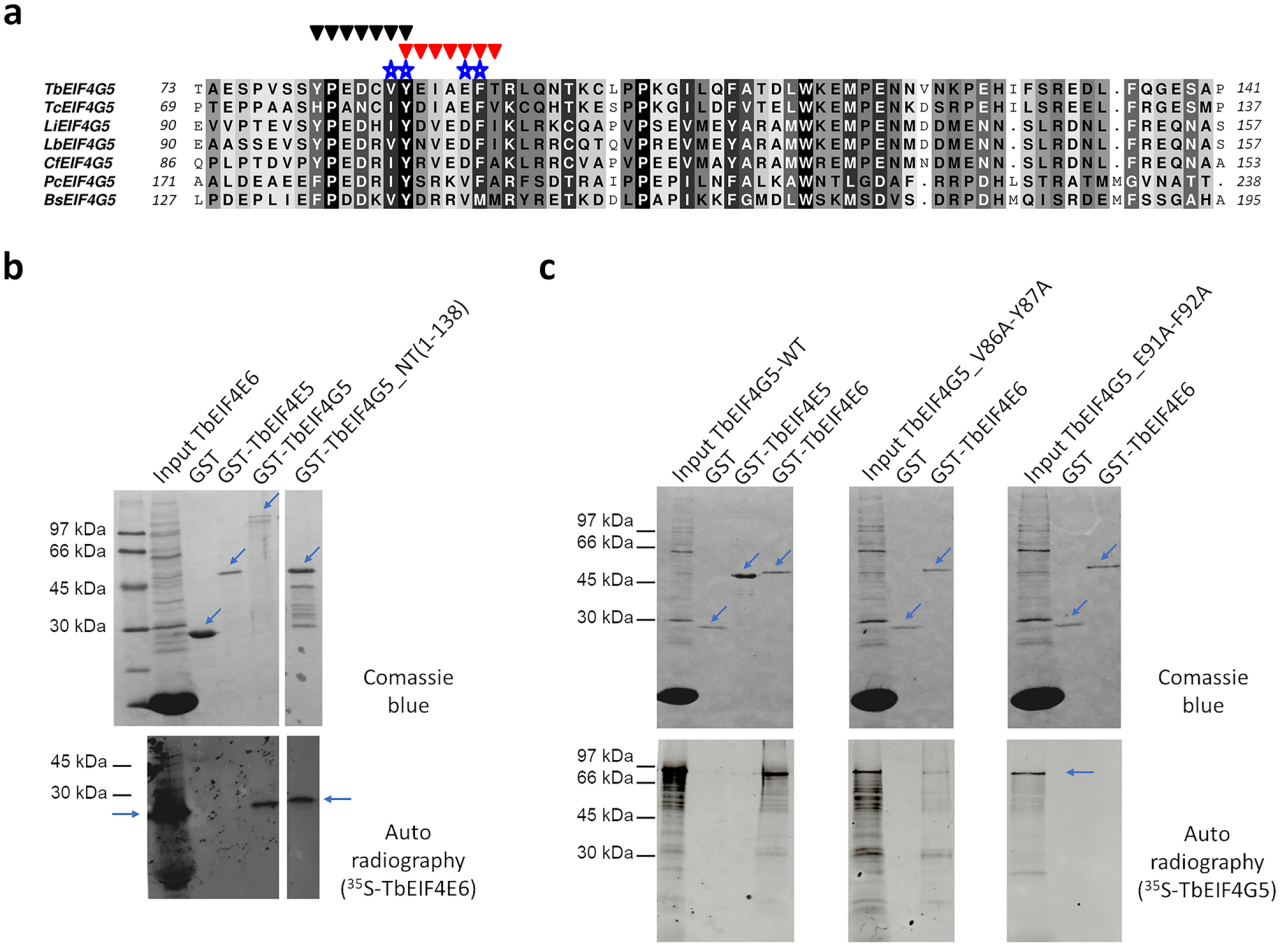
Analysis of EIF4E6 binding motifs within *T. brucei* EIF4G5. (**a**) Sequence alignment of a conserved 60-residue segment at the N-terminal portion of trypanosomatid EIF4G5 orthologues (*Trypanosoma brucei* (Tb), *T. cruzi* (Tc), *Leishmania infantum* (Li), *L. braziliensis* (Lb), *Crithidia fasciculata* (Cf), *Paratrypanosoma confusum* (Pc) and *Bodo saltans* (Bs)). The two sets of black and red triangles indicate two motifs conserved among the analyzed sequences that most closely resemble the previously defined canonical eIF4E binding motif (YX_4_LΦ) within eIF4Gs. The stars indicate the two pairs of residues (86VY87) and (91EF92), which were replaced by alanines in this study. The background grayscale indicates the degree of residue conservation, from white (less conserved) to black (identical). (**b**) Analysis of the interaction of GST-tagged TbEIF4G5 and ^35^S-labelled TbEIF4E6 by pull-down assays using Glutathione Sepharose beads. Two GST-tagged TbEIF4G5 constructs were used in the assay, the full-length protein and the N-terminal region (TbEIF4G5_NT), comprising residues 1 to 138. GST and GST-TbEIF4E5 were used as negative controls. (**c**) Reciprocal pull-down assay carried out with GST-tagged TbEIF4E6 and ^35^S-labelled TbEIF4G5. Binding to TbEIF4E6 of the wild type (WT) TbEIF4G5 and the two TbEIF4G5 variants (V86A-Y87A) and (E91A-F92A) were assessed. GST and GST-TbEIF4E5 were also used as negative controls. In (**b**) and (**c**), the gels stained by Coomassie blue are shown in the top panels, with the relevant bands indicated by arrows. The lanes named Input TbEIF4E6 and Input TbEIF4G5 correspond to the in vitro/transcription/translation lysates, while GST and the GST fusion proteins were obtained from *E. coli* expression. The high abundance low molecular weight band observed in the input lanes corresponds to hemoglobin, present in the rabbit reticulocyte lysates used for the in vitro translation. The autoradiographs are shown on the bottom. Full images of the gels and autoradiographs are shown in Supplementary Fig. 3.

Regarding the EIF4E6 binding region, supposedly located at the N-terminal portion of EIF4G5 as for all other known eIF4Gs, a conserved segment of approximately 60 residues was identified, which includes several aromatic residues that could be involved in the interaction (Fig. 1a). The two motifs that more closely resemble the canonical YX_4_LΦ motif within this segment are 81-YPEDCVY-87 and 87-YEIAEFT-93, which conserve an aromatic and a hydrophobic residue in the first and sixth positions, respectively, in all kinetoplastid EIF4G5 sequences analyzed.

To investigate the TbEIF4E6 binding region, we carried out pull-down assays first comparing the GST-tagged full-length TbEIF4G5 with a fragment corresponding to its N-terminal region (residues 1–138), which comprises the two abovementioned segments. Both the full-length and the N-terminal segment of TbEIF4G5 were found to bind to the ^35^S-labelled TbEIF4E6, confirming the localization of the TbEIF4E6 binding region within the TbEIF4G5 N-terminal segment (Fig. 1b). Reciprocal pull-down assays were then set up with GST-tagged TbEIF4E6 tested for its ability to bind to ^35^S-labelled TbEIF4G5. In addition to wild-type TbEIF4G5, two variants were produced, V86A-Y87A and E91A-F92A, mutating sets of two consecutive residues which we believed most likely could affect the TbEIF4E6 interaction and which were localized within the partially conserved motifs resembling the canonical eIF4E binding motif. As expected, binding of wild-type TbEIF4G5 to TbEIF4E6 was confirmed. On the other hand, V86A-Y87A substitutions had an impact on the interaction, whereas E91A-F92A substitutions abolished the binding between the two proteins (Fig. 1c). These results indicate the participation of these and surrounding residues in mediating the TbEIF4E6-4G5 interface.

### Interaction with the TbEIF4G5_79-116 peptide promotes remarkable stabilization of TbEIF4E6

**Figure 2:**
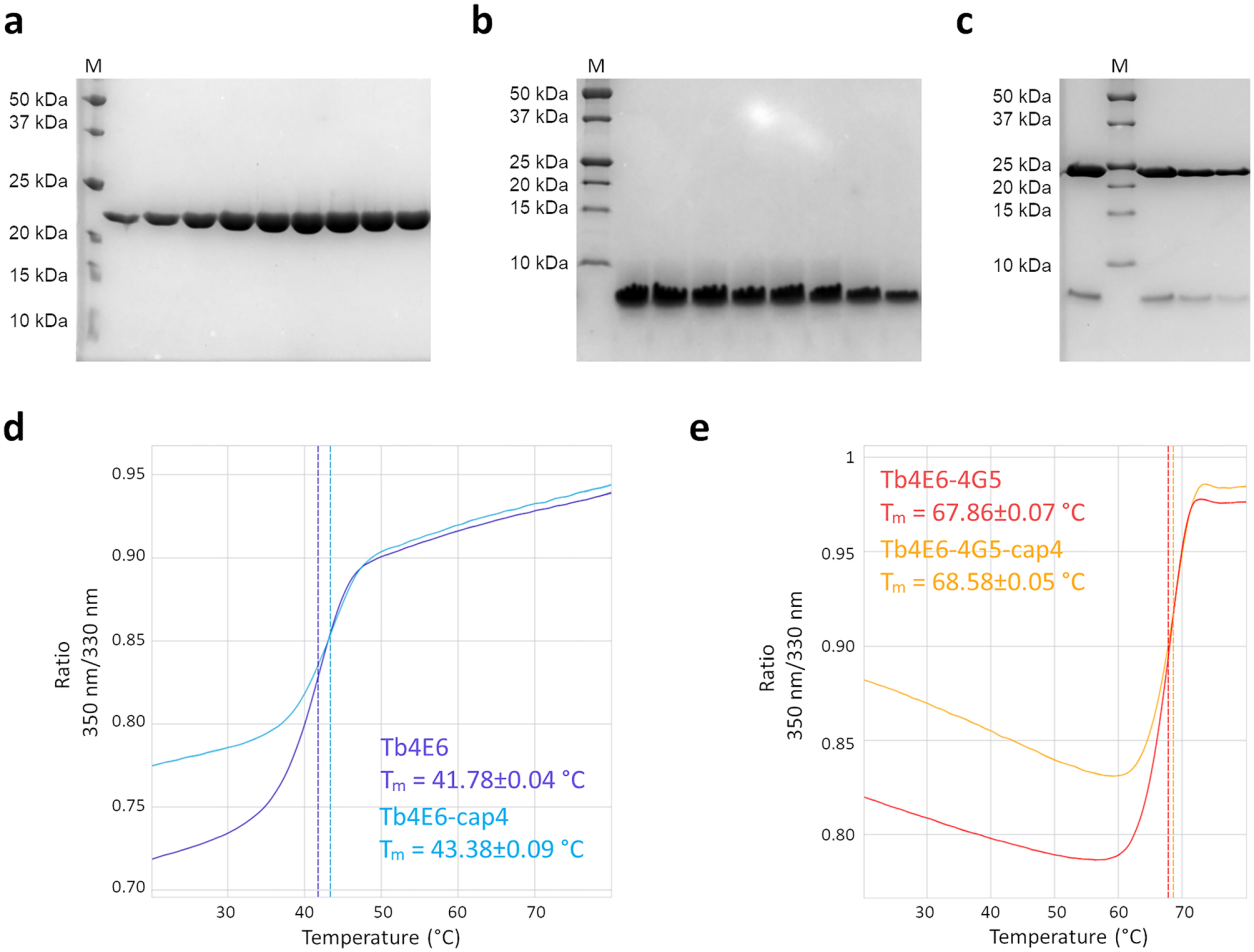
Purification and thermal stability analysis of TbEIF4E6 and its complex with the TbEIF4G5_79-116 peptide. (**a**–**c**) SDS‒PAGE analysis of purified recombinant *T. brucei* EIF4E6 (**a**), TbEIF4G5_79-116 peptide (**b**) and the TbEIF4E6-4G5 complex (**c**). The molecular masses of the recombinant proteins were 21.4 kDa (TbEIF4E6) and 4.8 kDa (TbEIF4G5_79-116). The masses of the molecular marker (M) are indicated on the left-hand side. (**d**–**e**) Thermal unfolding analysis performed by nanoDSF. The ratio of emission intensity at 350 nm and 330 nm wavelengths is plotted as a function of temperature, and the transition temperature (Tm) is determined by the inflection point. (**d**) Thermal unfolding analysis of TbEIF4E6 prior to and after incubation with cap-4 (dark and light blue, respectively). (**e**) Thermal unfolding analysis of the TbEIF4E6-4G5 complex prior to and after incubation (red and orange, respectively). Tm values are indicated in the figure.

Based on sequence analysis, mutagenesis assays and the crystal structure of the human eIF4E-eIF4G complex^8^**,** we defined the TbEIF4G5 fragment comprising residues 79 to 116 to be expressed and purified for interaction and cocrystallization assays with TbEIF4E6. This fragment, 79-SSYPEDCVYEIAEFTRLQNTKCLPPKGILQFATDLWKE-116, includes the two motifs (underlined) investigated in the mutagenesis assays. TbEIF4E6 and the TbEIF4G5_79-116 peptide were produced in *E. coli* in high yields and were both successfully purified, and the TbEIF4E6-4G5 complex was successfully reconstituted in vitro (Fig. 2).

The stability of TbEIF4E6 in the apo form, in the presence of the TbEIF4G5_79-116 peptide and of the cap-4 analogue was evaluated by nano differential scanning fluorimetry (nanoDSF). This analysis showed that binding of the TbEIF4G5_79-116 peptide markedly increased the TbEIF4E6 melting temperature by more than 25 °C (Fig. 2). In contrast, the presence of the cap-4 analogue did not significantly change TbEIF4E6 stability. This result indicates a highly stable association between TbEIF4E6 and TbEIF4G5.

### The crystal structure of the *T. brucei* EIF4E6-4G5 complex reveals an extensive interaction surface

**Figure 3:**
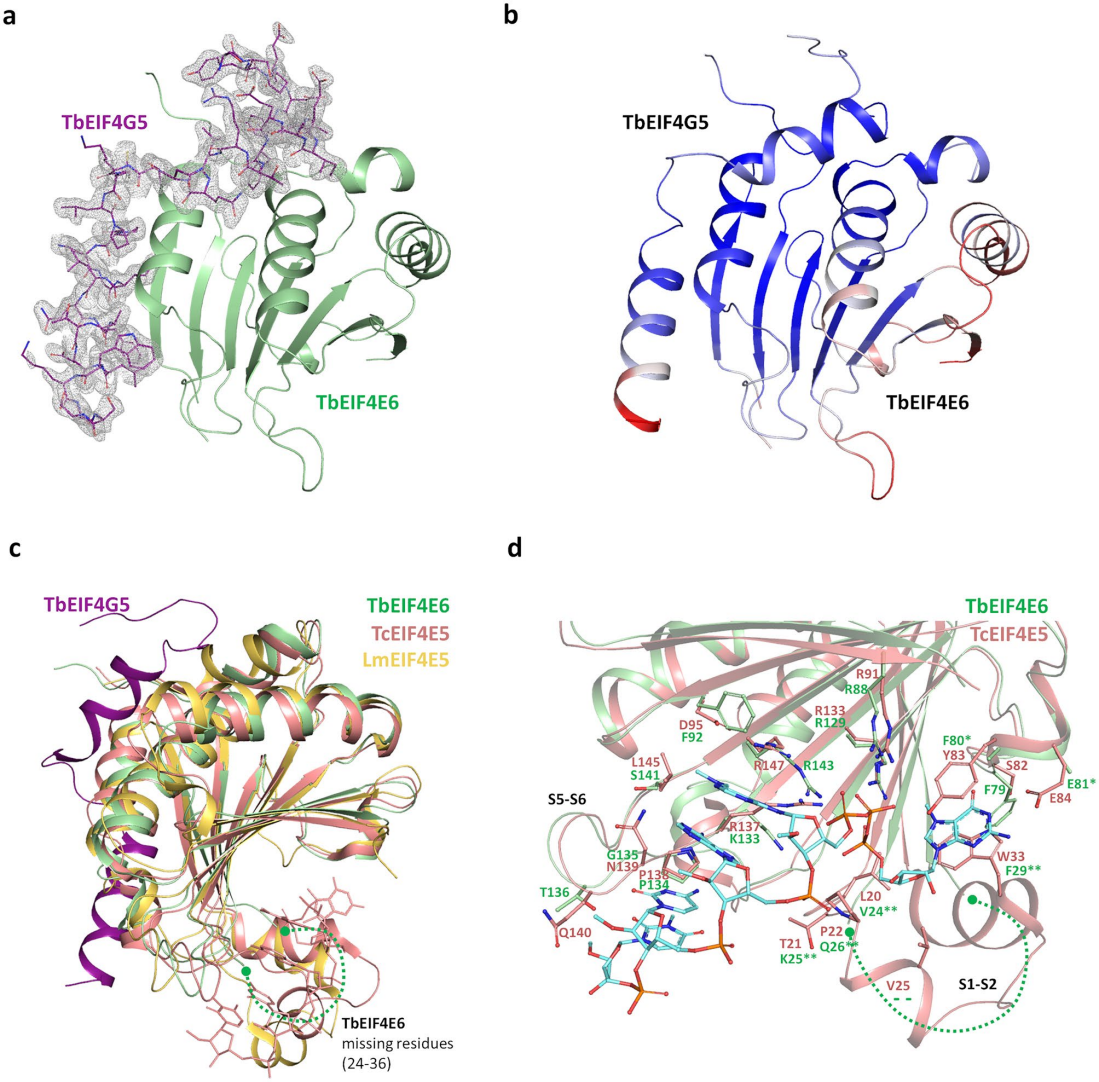
Crystal structure of the *T. brucei* EIF4E6-4G5 complex. (**a**) The structure of TbEIF4E6 is represented in green ribbons, and the TbEIF4G5_79-116 peptide is shown in purple sticks. The 2mFo-DFc electron density map corresponding to the TbEIF4G5_79-116 peptide is shown in gray and contoured at 1.1 sigma. (**b**) Model of TbEIF4E6-4G5 colored according to the B-factor values, from blue to red indicating increasing B-factor. (**c**) Superposition of the *T. brucei* EIF4E6-4G5 complex (4E6 and 4G5-peptide are shown in green and purple, respectively), *L. major* EIF4E5 (yellow, PDB 7KCJ) and *T. cruzi* EIF4E5-cap4 complex (salmon, PDB 6O7Y). The cap-4 analogue bound to TcEIF4E5 is shown in salmon sticks. The β1–β2 connecting region, which is missing in the TbEIF4E6 crystallographic model (residues 24–36), is indicated by a dashed line. (**d**) Detailed analysis of the cap-4 interacting region. The *T. cruzi* EIF4E5 structure with bound cap-4 analogue is represented in salmon with cap-4 shown in blue sticks. Cap-4 interacting residues in TcEIF4E5 and corresponding residues in TbEIF4E6 (shown in green) are represented in sticks and labeled. Residues lacking their side chains in the crystallographic model (F80, E81) are highlighted with a single star, whereas residues indicated by a double star are in the missing β1–β2 connecting region (S1–S2). The loop connecting β5 and β6 strands (S5-S6) which also participates in the cap-4 interaction is indicated.

The structure of *T. brucei* EIF4E6 in complex with the TbEIF4G5_79-116 peptide (hereafter called TbEIF4E6-4G5) was determined by molecular replacement using the EIF4E6 model predicted by AlphaFold^34^ as the search model. The TbEIF4E6-4G5 structure was refined at 1.90 Å resolution to a final R_work_/R_free_ of 0.1923/0.2219 (Table 1). The final model comprises residues 7 to 178 of TbEIF4E6 (out of 186), with the polypeptide chain clearly defined by the electron density. The exception is the region comprising residues 24–36, which could not be modelled. The electron density maps also clearly showed the TbEIF4G5_79-116 peptide (Supplementary Fig. 4), which could be entirely modelled (residues 79 to 116, plus two residues from the expression vector) (Fig. 3a). In contrast, even though the TbEIF4E6-4G5 complex was incubated with the cap-4 analogue prior to crystallization, no electron density corresponding to cap-4 was observed during refinement of the structure, indicating that it did not bind to TbEIF4E6 under the conditions tested. Analysis of B-factor distribution in the TbEIF4E6-4G5 structure showed very similar average B values for the EIF4E6 (36.63 Å^2^ overall) and EIF4G5 (37.47 Å^2^ overall) chains. The highest B values are observed around the cap-binding region in EIF4E6 and at the C-terminal end of the 4G5 peptide (Fig. 3b).

**Table 1:** Crystallographic data and refinement statistics.

Data statistics	
Source	SOLElL-PX2A
Wavelength (Ȧ)	0.978565
Resolution (Ȧ)	44–1.90 (1.97- 1.90)
Space group	C222_1_
Unit cell (Ȧ)	a = 65.50 b = 131.18 c = 59.66
Number of observations	218,945 (10,788)
Number of unique reflections	16,430 (822)
Completeness spherical (%)	79.2 (36.2)
Completeness ellipsoidal (%)^#^	95.1 (85.9)
Multiplicity	13.3 (13.1)
Mean(I)/sd(I)	13.2 (2.1)
CC½	99.5 (54.2)
Refinement statistics	
R_work_	0.1923
R_free_	0.2219
Bond RMSD Length(Ȧ)/ Angle (°)	0.008/0.90
B values (Ȧ^2^)	
From Wilson Plot	24.80
Mean B value (overall)	37.26
Number of protein atoms	1557
Number of solvent atoms	120
Ramachandran plot	
Favored (%)	98.45
Outliers (%)	0

Values in parentheses are for the highest resolution shell. ^#^Diffraction limits and principal axes of the ellipsoid fitted to the diffraction cutoff surface: a* = 2.40; b* = 1.88; c* = 1.78. Criteria used in determination of diffraction limits: Local mean I/σ(I) ≥ 1.20.

As expected, comparison with eIF4E homologues confirms the overall structural conservation. Structural superposition of TbEIF4E6 with the *T. cruzi* EIF4E5 homologue in complex with mRNA cap-4 (PDB 6O7Y^31^) resulted in an RMSD of 1.52 Å for 153 aligned C-alpha atoms. Alignment of TbEIF4E6 and *L. major* EIF4E5 in the apo form (PDB 7KCJ^32^) resulted in an RMSD of 1.55 Å for the aligned 153 C-alpha atoms (Fig. 3c). Interestingly, the absence of electron density in the region connecting TbEIF4E6 β1–β2 strands (residues 24–36), which in TcEIF4E5 was shown to be largely involved in the interaction with cap-4^31^, indicates its conformational flexibility in the TbEIF4E6-4G5 structure. This result contrasts with what was observed in the *L. major* EIF4E5 structure, where even in the absence of cap-4, the S1–S2 region was well defined and adopted an open conformation (Fig. 3c and d).

A detailed analysis of the cap-4 interacting region shows the conservation of the essential characteristics of the main residues involved in binding (Fig. 3d). Although the flexibility of the TbEIF4E6 S1-S2 region and of the side chains of surrounding residues prevents the complete analysis of the cap-binding pocket, the two aromatic residues F29 and F80 and the adjacent E81 potentially maintain the ability to form the classical interaction with the m^7^G moiety, with F29 and F80 forming the m^7^G sandwich and the highly conserved glutamic acid participating in polar interactions with the guanine base. The basic side chains involved in interactions with the triphosphate are also conserved, whereas some nonconservative substitutions of residues involved in the interaction with the AACU nucleotides are observed (Fig. 3d).

**Figure 4:**
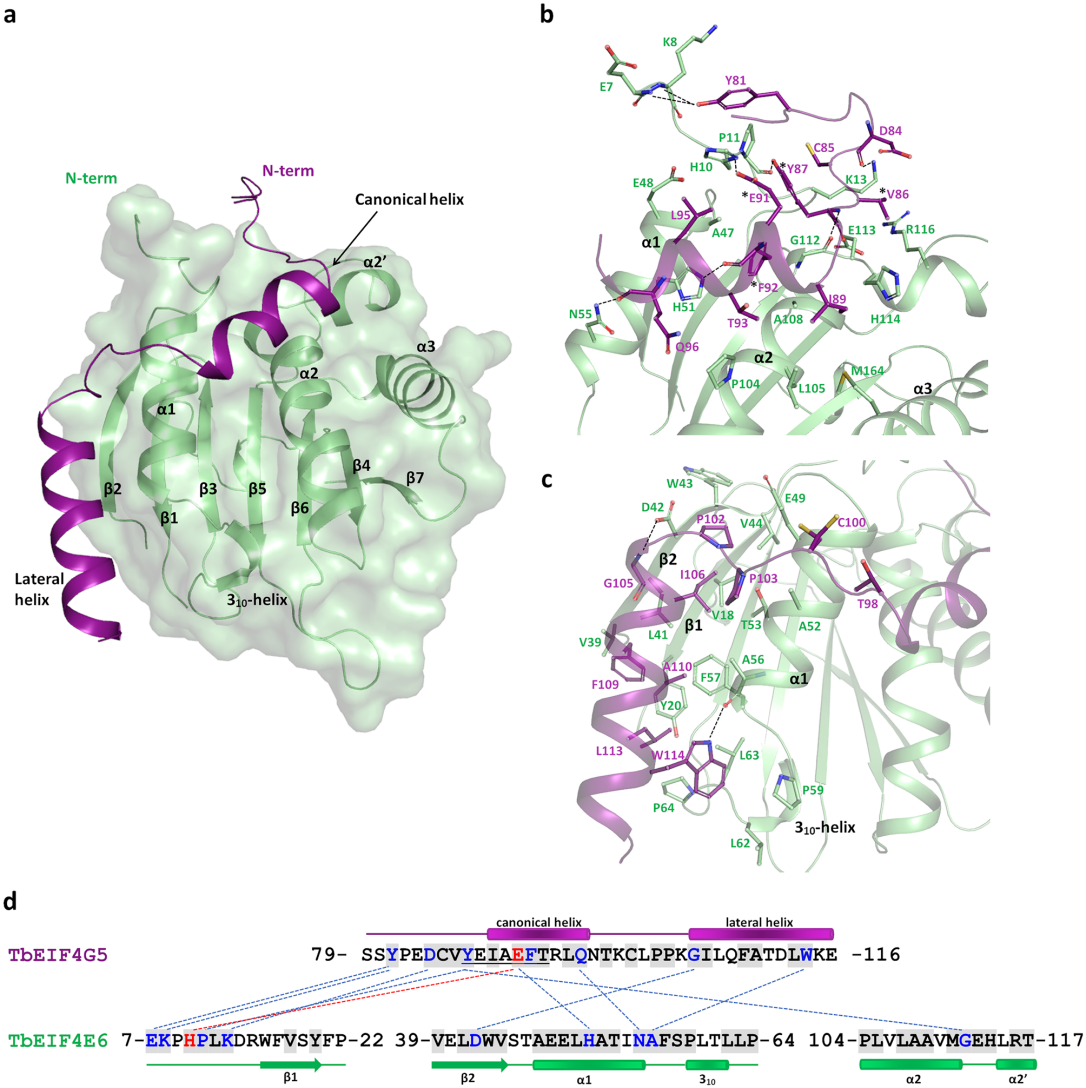
Description of the *T. brucei* EIF4E6/TbEIF4G5_79-116 complex interface. (**a**) TbEIF4E6-4G5 overall structure, showing TbEIF4E6 in green and the TbEIF4G5_79-116 peptide in purple. The EIF4G5 canonical and lateral helices and EIF4E6 secondary structure elements are labelled. (**b** and **c**) Details of the EIF4E6-4G5 interaction. The main residues involved in the interaction are shown in sticks and labelled. Hydrogen bonds are indicated by dashed lines. The interaction region comprising the TbEIF4G5 N-terminal and canonical helix is shown in (**b**). The binding region involving the TbEIF4G5 loop and lateral helix is shown in (**c**). The TbEIF4G5 residues mutated in this work are identified with an asterisk. (**d**) TbEIF4G5 and 4E6 interacting residues mapped in the primary structures. Residues participating in hydrogen bonds and salt bridges are colored blue and red, respectively, with dashed lines connecting them. The gray background indicates the residues that have more than 20% of their total area buried by the complex interface. The partially conserved sequence equivalent to the eIF4G canonical YX_4_LΦ motif is underlined. The secondary structure elements are indicated on the top/bottom of the sequences.

The TbEIF4E6-4G5 complex shows an extensive interaction surface (Fig. 4a). The TbEIF4E6-4G5 association involves 10 hydrogen bonds and one salt bridge. Moreover, 21 out of the 38 residues of the TbEIF4G5_79-116 peptide cocrystallized with TbEIF4E6 had 20% or more of their total area buried by the interface. From the TbEIF4E6 side, 39 residues have 20% or more of their area buried by the interface. Several residues from the N-terminal region of TbEIF4E6 interact with the N-terminal of the TbEIF4G5_79-116 peptide, which includes the tyrosine residue (Y87) from the partially conserved canonical motif. The TbEIF4G5 canonical helix interacts mainly with TbEIF4E6 residues from helices α1 and α2 through both polar and hydrophobic contacts (Fig. 4b). The loop connecting the two TbEIF4G5_79-116 peptide helices, together with the lateral helix, also accounts for a large number of contacts with TbEIF4E6 residues from helix α1 and strands β1 and β2, including a hydrogen bond between tryptophan 114 located at the C-terminal region of the TbEIF4G5 lateral helix and the carbonyl group of alanine 56 from TbEIF4E6 α1 (Fig. 4c).

Our mutagenesis assays revealed that replacement of TbEIF4G5 V86/Y87 by alanines has an impact on TbEIF4E6-4G5 binding, whereas substitution of TbEIF4G5 E91/F92 completely abolishes the interaction. These findings are consistent with the structural data. Tyrosine 87 is involved in a hydrogen bond with the main chain carbonyl group of TbEIF4E6 proline 11, and valine 86 is buried by the complex interface. Glutamic acid 91 forms a salt bridge with TbEIF4E6 N-terminal His10, and the main chain carbonyl group of F92 is involved in a hydrogen bond with TbEIF4E6 His51 (from helix α1) (Fig. 4b). Moreover, the bulky side chain of F92 is completely buried in the complex interface, participating in hydrophobic interactions.

### *T. brucei* EIF4E6-4G5 interface reveals distinct features compared with the human counterparts

**Figure 5:**
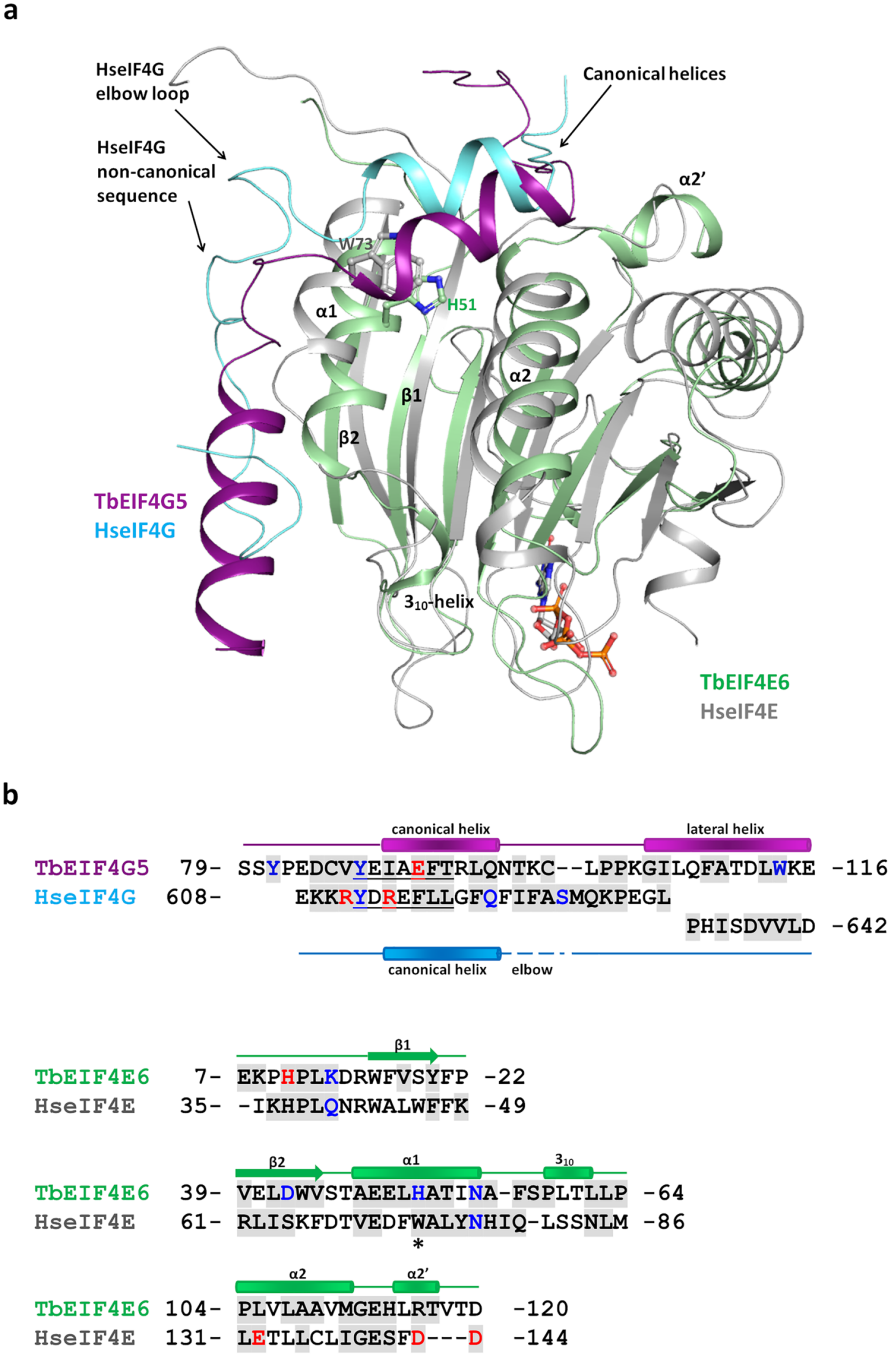
Structural comparison between *T. brucei* EIF4E6-4G5 and human eIF4E-4G complexes. (**a**) Overall superposition of the complexes. Human eIF4E and 4G (PDB 5T46) are represented in gray and cyan, respectively. TbEIF4E6 and TbEIF4G5_79-116 are shown in green and purple, respectively. The m^7^GTP analogue bound to human eIF4E is represented in sticks. The side chains of the conserved eIF4E tryptophan residue (W73) and the corresponding H51 in TbEIF4E6 are shown in sticks and labelled. The TbEIF4E6 secondary structure elements that participate in the interaction are labelled. (**b**) Structure-based sequence alignment of eIF4G and 4E interacting residues. The residue numbering of the segments is indicated. The eIF4G motif YXXXXLΦ and corresponding sequence in TbEIF4G5 are underlined. The C-terminal end of the HseIF4G peptide shown in the lower line does not align with the TbEIF4G5 lateral helix. Residues participating in hydrogen bonds via their side chains and those forming salt bridges are colored blue and red, respectively. The gray background indicates the residues that have more than 20% of their total area buried by the complex interface. EIF4E H51/W73 are indicated by an asterisk.

Structural alignment of TbEIF4E6-4G5 with human eIF4E (HseIF4E) in complex with the eIF4G interacting peptide (PDB 5T46^8^**)** resulted in an RMSD of 2.26 Å for 165 C-alpha atoms aligned, 24 of which were from the 4G peptide. Despite the global match of the human eIF4G and TbEIF4G5 regions binding to eIF4E, with superposition of the canonical helices, significantly distinct features are observed (Fig. 5). The TbEIF4G5 lateral helix replaces the coil structure adopted by the noncanonical interacting sequence from human eIF4G (Fig. 5a). The connection between helix α2 and strand β5 of TbEIF4E6 has a three-residue insertion when compared with HseIF4E, forming a short helix that we identified as α2′. This short helix forms a distinct interface with the EIF4G5 residues at the N-terminus of the canonical helix (Fig. 5a). The human eIF4E-4G complex is further stabilized by salt bridges that are not conserved in the TbEIF4E6-4G5 complex. Human eIF4E Glu132 (from helix α2) interacts with eIF4G Arg614 (from the canonical helix) and eIF4E Asp143/144 (from the α2-β5 loop) with eIF4G Arg611 (from the loop at the N-terminal side of the canonical helix). The only residues involved in polar interactions, which are conserved in *T. brucei* and human counterparts, are TbEIF4E6 N55 (human eIF4E N77) and TbEIF4G5 Y87 (human eIF4G Y612, belonging to the canonical motif) (Fig. 5b).

Interestingly, the generally conserved eIF4E tryptophan residue (W73 in human eIF4E), which was shown to be essential for the interaction with eIF4G^17,37,38^, is replaced by a histidine (H51) in TbEIF4E6 (Fig. 5). The equivalent residue in EIF4E6 from *T. cruzi*, *Leishmania* species and *C. fasciculata* is a tyrosine whereas EIF4E6 from more divergent trypanosomatids *P. confusum* and *B. saltans* conserves a tryptophan residue. To investigate the impact of the H51 substitution on the TbEIF4E6-4G5 interaction, a mutagenesis experiment was performed by replacing histidine 51 with alanine. The TbEIF4E6-H51A mutant was successfully expressed in *E. coli,* and its complex with the recombinant TbEIF4G5_79-116 peptide was successfully reconstituted (Supplementary Fig. 5), showing that this mutation did not abolish complex formation, possibly due to the large areas of the interacting surfaces, where many other residues contribute to the interface (Fig. 4). However, the H51A mutation does seem to affect the stability of the complex, as determined by thermal denaturation assays. While TbEIF4E6-H51A showed a melting temperature similar to that of wild-type TbEIF4E6, the TbEIF4E6-H51A/TbEIF4G5_79-116 complex showed two transition points, indicating partial dissociation of the complex (Supplementary Fig. 5). In addition, the melting temperature of the complex containing the TbEIF4E6-H51A mutant was approximately 4 °C lower than the melting temperature of the complex containing wild-type TbEIF4E6 (compare Fig. 2 with Fig. S5), which is consistent with the role of H51 as a component of the interacting surface.

**Figure 6:**
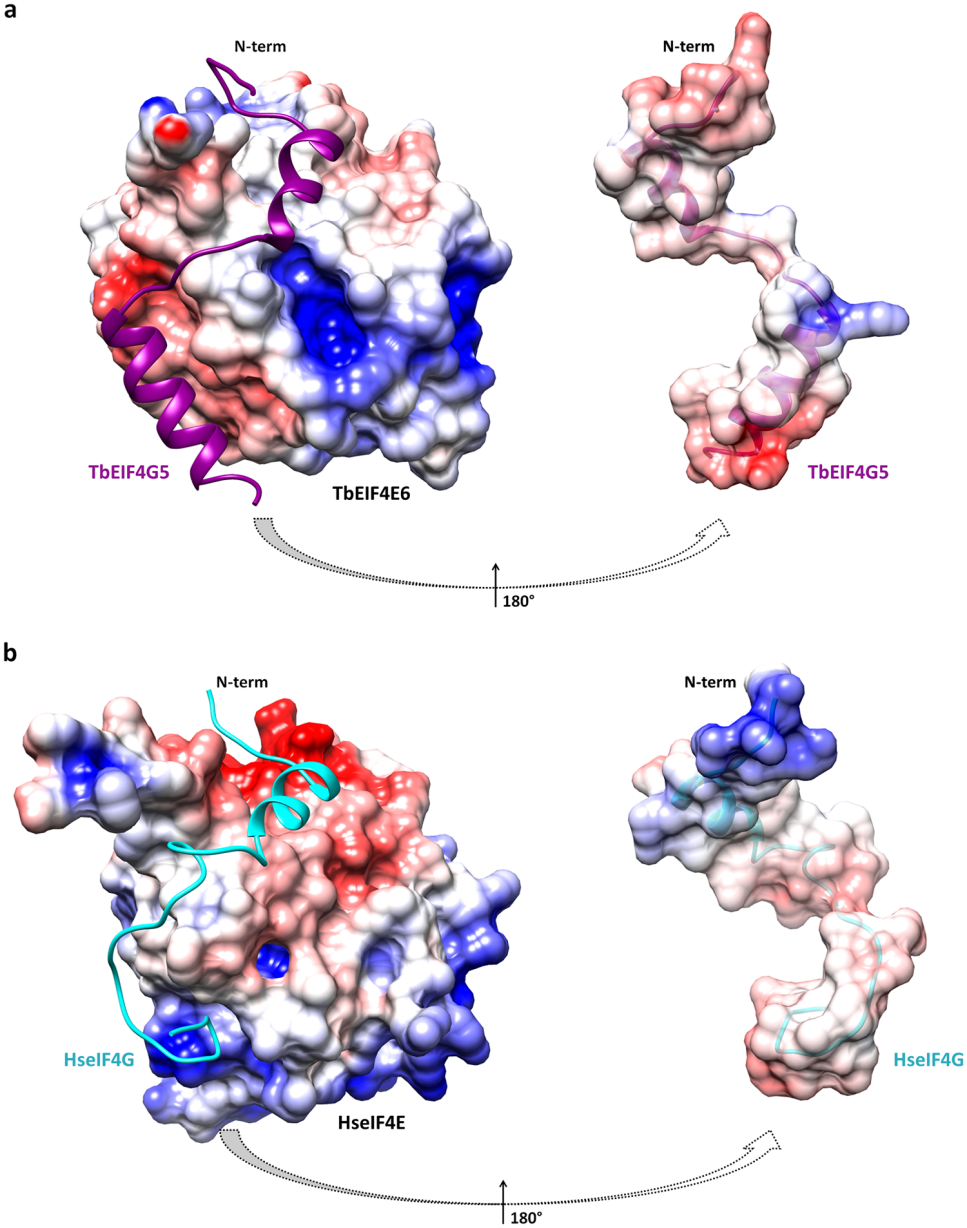
Comparison of the electrostatic potential surfaces of *T. brucei* EIF4E6-4G5 (a) and human eIF4E-4G (b) complexes. The left panels show the electrostatic potential surfaces of eIF4E with the 4G fragments represented in ribbons. The right panels show the electrostatic potential surfaces of the 4G peptides. The 4G peptide models are turned by 180 degrees relative to the orientation shown on the left to display their interaction surface. The boundaries for potential contour map visualization are − 5 kT/e (red) and + 5 kT/e (blue).

A comparative analysis of electrostatic potential surfaces depicts differences in charge distribution along the interface of the human eIF4E-4G and TbEIF4E6-4G5 complexes (Fig. 6). The N-terminus of the TbEIF4G5_79-116 peptide shows a net negative charge distribution that correlates with the positive charge of the TbEIF4E6 interacting region (Fig. 6a). The opposite is observed when analyzing the N-terminal region of the human 4G peptide/eIF4E interaction (Fig. 6b). The interfacing surface of the 4G canonical helices and noncanonical regions are predominantly hydrophobic in both complexes, except for the N-terminal end of the TbEIF4G5 lateral helix. This region shows a positive charge distribution, correlating with a more marked negative charge in the corresponding TbEIF4E6 interacting region. Despite the overall structural conservation of the eIF4E domains and of their global surfaces engaged in eIF4G binding, specific structural features differentiate each pair of eIF4E-4G homologues. Additional comparative analysis with eIF4E-4G structures from *Drosophila melanogaster*^8^ and *Chaetomium thermophilum*^9^ underpins the unique characteristics of each interaction interface (Supplementary Fig. 6).

### Distinctness between *T. brucei* EIF4E6-4G5 and *L. major* EIF4E1-4EIP interfaces

**Figure 7:**
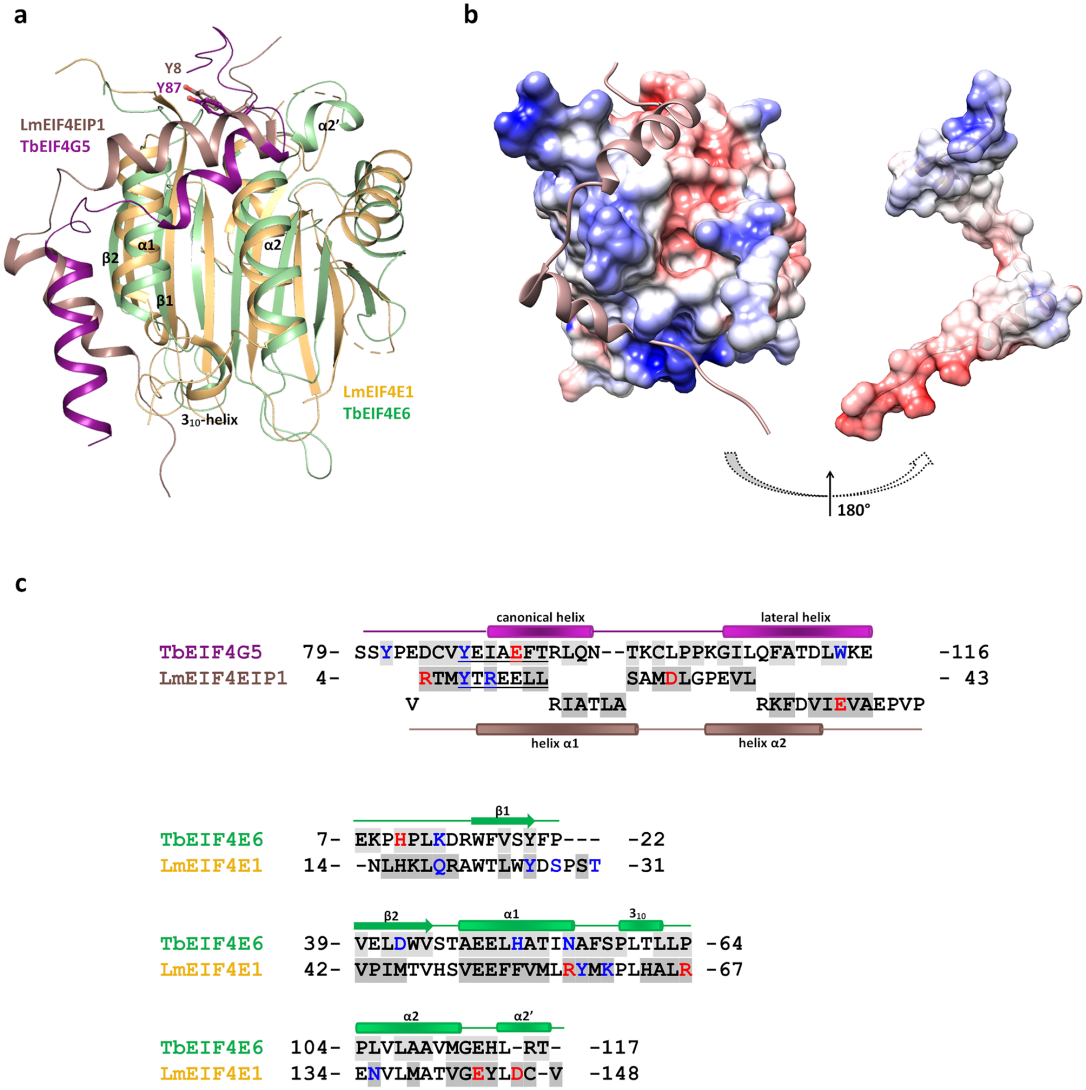
Structural comparison between *T. brucei* EIF4E6-4G5 and *L. major* EIF4E1-4EIP1 complexes. (**a**) Overall superposition of the complexes. *L. major* EIF4E1 and 4EIP1 (PDB 5WB5) are represented in gold and brown, respectively. TbEIF4E6 and TbEIF4G5_79-116 are shown in green and purple, respectively. The side chains of the TbEIF4G5 tyrosine residue (Y87) of the eIF4G canonical motif and the corresponding Y8 in LmEIF4EIP1 are shown in sticks and labelled. The TbEIF4E6 secondary structure elements that participate in the interaction with 4G5 are labelled. (**b**) Electrostatic potential of LmEIF4E1-4EIP1. The left panel show the electrostatic potential surface of LmEIF4E1 with the 4EIP1 fragment represented in ribbon. The right panel show the electrostatic potential surface of 4EIP1. The peptide model is turned by 180 degrees relative to the orientation shown on the left to display its interaction surface. The boundaries for potential contour map visualization are − 5 kT/e (red) and + 5 kT/e (blue). (**c**) Structure-based sequence alignment of TbEIF4G5 and 4E6 interacting residues with LmEIF4EIP1 and 4E1, respectively. The residue numbering of the respective segments is indicated. The eIF4G motif and corresponding sequence in LmEIF4EIP1 are underlined. The sequences of the 4EIP1 peptide shown in the lower line does not align with TbEIF4G5. Residues participating in hydrogen bonds and those forming salt bridges are colored blue and red, respectively. The gray background indicates the residues that have more than 20% of their total area buried by the complex interface.

Trypanosomatids EIF4E1 and EIF4E2 are distinguished from other homologues since so far they were not shown to bind eIF4G-like scaffold proteins^17,18,26^. However, a *Leishmania* EIF4E1 interacting protein (Leish4EIP1) containing a YXXXXLΦ motif near its N terminus was identified^17^.The three-dimensional structure of *L. major* EIF4E1 in complex with a fragment of Leish4EIP1 was already described, revealing that this fragment adopts the helical eIF4E binding consensus structure, followed by a flexible linker and a second α-helix^30^. Structural alignment of *T. brucei* EIF4E6-4G5 complex and *L. major* EIF4E1-4EIP1 (PDB 5WB5^30^) resulted in an RMSD of 2.34 Å for 158 C-alpha atoms aligned, 19 of which from the 4EIP1 peptide. Despite de global conservation of the binding mode, distinct features are observed (Fig. 7). Lm4EIP1 shows a longer helix α1 when compared to 4G5 canonical helix whereas helix α2 is shorter than the 4G5 lateral helix (Fig. 7a, c). Analysis of the electrostatic potential surfaces reveals a distinct charge distribution in the LmEIF4E1-4EIP1 interface when compared with TbEIF4E6-4G5 (Fig. 7b compared to Fig. 6a). Moreover, the orientation of Lm4EIP1 helix α2 approaches its C-terminal end to the groove formed between LmEIF4E1 helix α1 and strand β1, in contrast to the Tb4G5 lateral helix whose orientation is more parallel to TbEIF4E6 helix α1. Structure-based sequence alignment of the interaction regions (Fig. 7c) shows low conservation of the residues directly implicated in binding on both eIF4E and 4E partner sides, reinforcing that specific molecular features are likely to determine the selectivity between pairs for the formation of eIF4E complexes.

## Discussion

**Figure 8:**
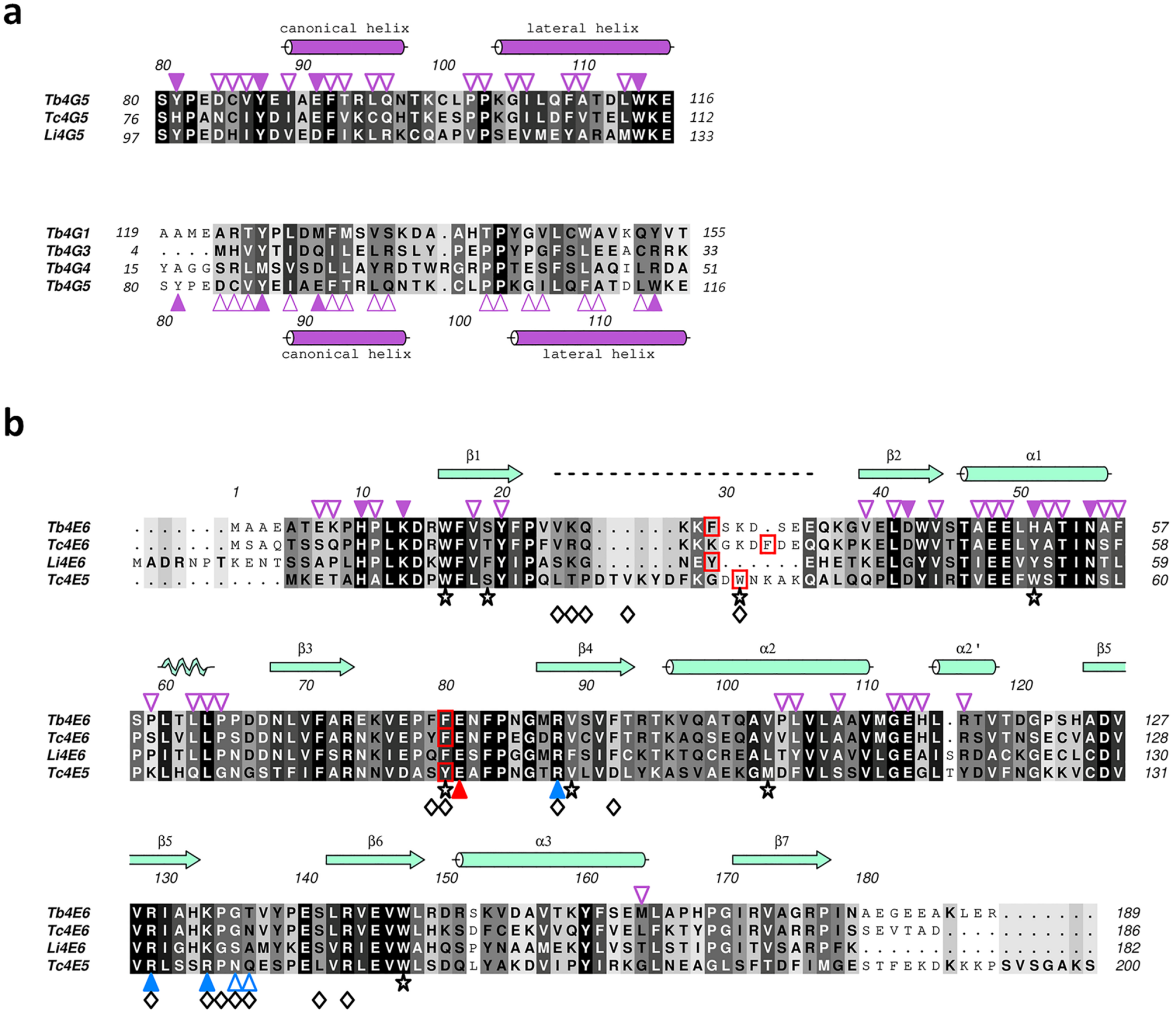
Structure-based sequence alignment of EIF4G5 and EIF4E6 homologues. (**a**) On the top: alignment of the fragment of TbEIF4G5 comprising the EIF4E6 interacting peptide *of T. brucei*, *T. cruzi* and *L. infantum* EIF4G5. On the bottom: alignment of the corresponding sequences of *T. brucei* EIF4G1, 4G3, 4G4 and 4G5. The secondary structure elements correspond to *T. brucei* EIF4G5. The purple triangles indicate the TbEIF4G5 residues involved in the interaction with TbEIF4E6 either by hydrophobic contacts (empty triangles) or by hydrogen bonds via their side chains (filled triangles). (**b**) Sequence alignment of EIF4E6 from *T. brucei* (Tb4E6), *T. cruzi* (Tc4E6), *L. infantum* (Li4E6) and *T. cruzi* EIF4E5 (Tc4E5). The black stars on the bottom indicate the position corresponding to the eight tryptophan residues usually conserved in eIF4E family members. The secondary structure elements and residue numbers indicated on the top correspond to *T. brucei* EIF4E6. The dashed line indicates the residues that are missing in the Tb4E6 crystallographic model (24–36). The purple triangles on the top of the sequences indicate the Tb4E6 residues involved in the interaction with EIF4G5 either by hydrophobic contacts (empty triangles) or by hydrogen bonds via their side chains (filled triangles). The aromatic residues that would be in position to form the stacking interaction with the m^7^G moiety of the mRNA cap are highlighted by red boxes. The residues of *T. cruzi* EIF4E5 involved in polar and hydrophobic interactions with cap-4^31^ are highlighted by triangles and diamonds, respectively, on the bottom of the alignment. The red triangle indicates the highly conserved glutamic acid that forms a salt bridge with the m^7^G guanidine. The blue filled triangles indicate the basic side chains involved in TcEIF4E5 interactions with the m^7^GTP phosphate groups, and the blue empty triangles indicate the residues involved in additional interactions with the second and third bases of the cap-4 analogue.

The structural analyses of the *T. brucei* EIF4E6/EIF4G5_79-116 complex and the comparisons with eIF4E-eIF4G/4EIP pairs from humans and other species carried out in this work have unveiled specific features of eIF4E and eIF4G factors likely to be responsible for the selective association needed to form distinct eIF4F complexes. They highlight the requirement for both canonical and noncanonical elements from the TbEIF4G5 N-terminal region in mediating the interaction with TbEIF4E6 in a structural arrangement that is similar to those described for metazoan and yeast proteins that bind eIF4E^10^ and has also been found to be conserved for the eIF4E/eIF4G interaction in plants^39^. However, despite the conservation of this structural arrangement, the data described here further define specific complementary features within both TbEIF4E6 and TbEIF4G5 binding surfaces, which are clearly different from the binding surfaces observed for the human eIF4E-4G and *L. major* EIF4E1-4EIP1 pairs, illustrating the specificities that drive the interactions between the different eIF4E homologues and their respective binding partners. Consistent with this hypothesis, the fragment of TbEIF4G5 comprising the EIF4E6-interacting peptide shows sequence conservation within *Trypanosoma* and *Leishmania* species (Fig. 8a, top panel). However, variations in the sequence of the eIF4G canonical motif are observed among trypanosomatid EIF4G5s, especially in the homologues from more divergent *P. confusum* and *B. saltans* (Fig. 1a). On the TbEIF4E6 side, most of the residues involved in the EIF4G5 interaction are conserved in *T. cruzi* and *Leishmania* orthologues, although a few nonconservative substitutions are observed (Fig. 8b). Therefore, as expected, EIF4E6 orthologues are likely to conserve the interaction mechanism with EIF4G5. On the other hand, sequence comparison of the TbEIF4G5_79-116 peptide with the equivalent region of the 4G1, 4G3 and 4G4 homologues revealed nonconservative substitutions of the residues involved in the EIF4E6 interaction (Fig. 8a, bottom panel). These differences may account for the selective interaction between specific EIF4E-4G pairs in trypanosomatids.

Concerning the mechanism of interaction with cap-4, the residues involved in cap binding are only partially conserved in the *T. brucei* and *L. infantum* EIF4E6 homologues, as can be observed from a structure-based sequence alignment of these homologues with the *T. cruzi* EIF4E5-cap-4 complex^31^ (Fig. 8b). The asparagine and glutamine residues located at the β5–β6 loop, involved in TcEIF4E5 interactions with the second and third bases of cap4, are not conserved among the homologues. In addition, TcEIF4E5 R137 is replaced by lysine residues in EIF4E6 homologues. Although this is a conservative substitution, R137 participates in hydrogen bonds with two cap-4 base moieties and salt bridges with the m^7^GTP phosphate groups, and a lysine residue may not fulfil the same role in stabilizing cap binding. The β1–β2 connecting region, which contains one of the aromatics involved in the stacking interaction with the m^7^G moiety and is missing from the TbEIF4E6 crystallographic model due to its flexibility, is shorter in EIF4E6 homologues than in EIF4E5 homologues. This difference in length, as well as in residue composition, may also have an impact on cap binding.

Our biophysical analyses have shown that the association with the TbEIF4G5_79-116 peptide greatly stabilizes the TbEIF4E6 structure, indicating the formation of a highly stable complex that does not require binding to cap-4. In fact, the interaction of recombinant TbEIF4E6 with cap analogues remains an intriguing point. As observed in the thermal stability assays, the presence of the cap-4 analogue led to only a small change in the melting temperature both for TbEIF4E6 alone and for the TbEIF4E6/TbEIF4G5_79-116 complex. In addition, although the TbEIF4E6/TbEIF4G5_79-116 complex was crystallized in the presence of a cap-4 analogue, no corresponding electron density was observed in the cap-binding site of TbEIF4E6. The absence of cap-4 is also consistent with the flexibility of the β1-β2 loop (residues 24–36), which could not be modelled in the crystal structure. Furthermore, microscale thermophoresis assays performed in our laboratory did not produce conclusive results on the interaction between TbEIF4E6 and cap analogues (data not shown). Regarding this point, it is important to mention that a previous study showed that recombinant TbEIF4E6 was able to bind to cap analogues (m^7^GTP and cap-4) in vitro^24^, results that could not be reproduced in our laboratory. However, *Leishmania* EIF4E6 does not seem to bind to m^7^GTP either, at least when assessed for its ability to bind to a m^7^GTP column^25^, an approach that can efficiently recruit other *Leishmania* eIF4E homologues, such as EIF4E1, EIF4E4 and EIF4E5^25,40,41^. The lack of cap binding activity by the TbEIF4E6/TbEIF4G5_79-116 complex might indicate a requirement for full-length TbEIF4G5 for the complex to effectively bind to cap-4. Alternatively, cap binding activity might require a third protein partner, such as G5-IP, a known TbEIF4E6/TbEIF4G5 binding partner. G5-IP coprecipitates with both TbEIF4E6- and TbEIF4G5-tagged baits and has been shown to bind directly to TbEIF4G5. It is characterized by the presence of a C-terminal half consisting of a guanylyltransferase domain, which has been found in capping enzymes^24^. Its strong association with the TbEIF4E6/TbEIF4G5 complex might thus be a requirement for proper cap binding, possibly requiring the guanylyltransferase domain.

*Leishmania* EIF4E6 was proposed to act as a translation repressor since its overexpression in promastigotes leads to a reduction in global translation^25^. This is consistent with studies in *T. brucei* procyclic cells, where TbEIF4E6 depletion by RNAi leads to a flagellar detachment phenotype but does not impact overall translation or growth^24^. Nevertheless, a different profile is seen in *T. brucei* bloodstream forms, where TbEIF4E6 was found to be essential and its depletion inhibited translation^26^, with both TbEIF4E6/TbEIF4G5 subunits also found to stimulate the expression of a reporter mRNA in tethering assays^27,28^. Indeed, the specific recruitment of TbEIF4E6/TbEIF4G5 to abundant mRNAs encoding *T. brucei* variant surface glycoproteins (VSGs) has been proposed as a mechanism to mediate the efficient translation of these mRNAs in bloodstream cells^29^. Since VSGs are not expressed in procyclic cells, the *T. brucei* insect life stage that is equivalent to the *Leishmania* promastigotes, a possible explanation for these discrepancies would be for the EIF4E6/EIF4G5-based complex to be active in translation only during the mammalian stage of the trypanosomatid life cycle, a possibility that needs to be investigated further. Regulating the activity of the EIF4E6/EIF4G5 complex would thus be critical for proper survival, differentiation and even pathogenesis of different trypanosomatid species, and this may require means to control efficient binding and release of target mRNAs. Our structural data would be consistent with an intrinsic low binding of the complex to mRNAs, but which could be enhanced by its interaction with other proteins and/or by posttranslational modifications which need to be better defined.

In conclusion, our results constitute a significant contribution toward a detailed understanding of the molecular bases for the formation of the multiple eIF4F-like complexes found in trypanosomatids. These organisms can be considered excellent models for the study of such interactions due to the multiple sets of eIF4Es and eIF4Gs forming different eIF4F-like complexes that are simultaneously expressed and seem to be functionally distinct. They can thus help to define distinctive structural features that are needed for the formation of specific eIF4E-4G complexes. The plant system is especially noteworthy since it is also associated with two distinct eIF4F complexes that are simultaneously active in translation^42,43^. Inhibitors of the mammalian eIF4E-4G interaction have been proposed to have therapeutic value, especially when used against cancer cells, and have achieved some promising results^44^. The best characterized of these thus far, 4EGI-1, acts allosterically and does not bind to the eIF4E/eIF4G interface^45^. Other inhibitors of potential therapeutic interest can nevertheless be expected to interfere directly with this interaction by mimicking, for instance, the eIF4E binding motif found within eIF4G. Any desired specificity requires a detailed characterization of the interaction between selected complexes, as shown here for TbEIF4E6/TbEIF4G5.

## Methods

### Sequence alignment

The TriTrypDB accession numbers of the orthologues used in the sequence alignments are: *Trypanosoma brucei* (Tb) EIF4G5–Tb927.8.4500; *Trypanosoma cruzi* (Tc) EIF4G5–TcCLB.508989.90; *Leishmania infantum* (Li) EIF4G5–LINF_100017900; *Leishmania braziliensis* (Lb) EIF4G5–LbrM.10.1180; *Crithidia fasciculata* (Cf) EIF4G5–CFAC1_040019400; *Paratrypanosoma confusum* (Pc) EIF4G5–PCON_0065880; *Bodo saltans* (Bs) EIF4G5–BSAL_82590; *Trypanosoma brucei* (Tb) EIF4G1–Tb927.5.1490; *Trypanosoma brucei* (Tb) EIF4G3–Tb927.8.4820; *Trypanosoma brucei* (Tb) EIF4G4–Tb927.11.10560; *Trypanosoma brucei* (Tb) EIF4E6–Tb927.7.1670; *Trypanosoma cruzi* (Tc) EIF4E6–TcC4B63_8g546; *Leishmania infantum* (Li) EIF4E6–LINF_260007400; *Trypanosoma cruzi* (Tc) EIF4E5–TcC4B63_13g194. Multiple sequence alignments were performed using the Clustal Omega program^46^. Structure-based sequence alignments were generated using PROMAL3D^47^ and further edited manually. Figures 1a and 8 were produced using the program ALINE^48^.

### Plasmid constructs and cloning procedures

The full-length wild-type (WT) genes encoding TbEIF4E6 (Tb927.7.1670), TbEIF4G5 (Tb927.8.4500) and TbEIF4E5 (Tb927.10.5020) were amplified by standard PCR using *T. brucei* Lister 427 genomic DNA. The amplicons were first cloned into the pGEM-T Easy vector (Promega) and sequenced, and the TbEIF4E6 and TbEIF4G5 genes were further subcloned into the pET21a vector (Novagen) between the *Bam*HI/*Xho*I (TbEIF4E6) and *Hin*dIII/*Not*I (TbEIF4G5) restriction sites. TbEIF4E6_H51A, TbEIF4G5_V86A-Y87A, TbEIF4G5_E91A-F92A and TbEIF4G5_NT (residues 1–138) mutants were generated in the pET21a vector using the QuikChange II Site-Directed Mutagenesis Kit (Agilent) following the manufacturer’s instructions. The primers used for site-directed mutagenesis are listed in Supplementary Table 1. For the GST-tagging of the proteins used in the in vitro pull-down assays, the coding sequences of TbEIF4E5, TbEIF4E6, TbeIF4E6_H51A, TbEIF4G5, TbEIF4G5_V86A-Y87A, TbEIF4G5_E91A-F92A and TbEIF4G5_NT were subcloned between the *Bam*HI/*Xho*I or *Bam*HI/*Not*I sites of the pGEX4T3 vector (GE Healthcare). To produce recombinant proteins fused to a GST-cleavable tag, a modified pGEX4T3 was generated (pGEX4T3TEV2), encoding the GST domain and thrombin cleavage site followed by an added *Nde*I cloning site and the sequence encoding the tobacco etch virus (TEV) protease site (residues GSHMENLYFQG) prior to the original *Bam*HI site. This was achieved through the annealing of one pair of complementary oligonucleotides (also listed in Supplementary Table 1) and cloning into the *Bam*HI of pGEX4T3, with the correct orientation maintaining the new, 3′ end, *Bam*HI site. Sequences encoding TbEIF4E6, TbEIF4E6_H51A and a fragment corresponding to the N-terminal portion of TbEIF4G5 (1–138), PCR amplified and cloned first into the pGEM-T Easy vector, were then subcloned into the *Bam*HI-*Xho*I sites of pGEX4T3TEV2. For recombinant expression of the TbEIF4G5_79-116 peptide, the nucleotide sequence encoding the TEV protease recognition site followed by the sequence encoding residues 79 to 116 of *T. brucei* EIF4G5 was synthesized by Synbio Technologies (South Brunswick, USA) with codon optimization for *Escherichia coli* expression and inserted into restriction sites *Kpn*I and *Xho*I of vector pET32a (Novagen). This construction allows the expression of the TbEIF4G5_79-116 peptide with a His6-tagged thioredoxin (TRX) fusion, which can be removed by the TEV protease.

### In vitro pull-down assays

Pull-down assays were performed as previously described^21^ using Glutathione-Sepharose 4B beads (Cytiva Life Sciences, former GE Healthcare) and GST-tagged proteins expressed in *Escherichia coli*. GST and the different GST-tagged proteins were immobilized on the beads and assayed for their ability to bind to the ^35^S-labelled proteins. The ^35^S-labelled proteins TbEIF4E6, TbEIF4G5, TbEIF4G5_V86A-Y87A and TbEIF4G5_E91A-F92A were obtained by using in vitro transcription/translation rabbit reticulocyte lysates (Promega) supplemented with ^35^S-methionine (Perkin Elmer) in the presence of the cap analogue. For the in vitro transcription/translation reactions, the corresponding pET21a-derived plasmids were linearized with *Xho*I and *Not*I. The radioactive signals were detected on autoradiographic films exposed to SDS‒PAGE 15% gels containing pull-down samples. The complete images of the SDS-PAGEs and autoradiographs shown in Fig. 1 and c are shown in Fig. S3.

### Protein production for biophysical and crystallization assays

To produce recombinant TbEIF4E6, *Escherichia coli* BL21 (DE3) slyD^−^ cells^49^ carrying the plasmid pRARE2 (Novagen) were transformed with the pGEX4T3TEV2-TbEIF4E6 vector and grown at 37 °C in LB medium plus the selection antibiotics. Expression was induced at 18 °C (OD_600_ ~ 0.7) for sixteen hours with 0.25 mM isopropyl-β-D-thiogalactopyranoside (IPTG). Cells from 2 L culture were harvested by centrifugation, resuspended in 20 mL of buffer A (140 mM NaCl, 2.7 mM KCl, 10 mM Na_2_HPO_4_, 1.8 mM KH_2_PO_4_, 5 mM DTT, pH 7.3, supplemented with EDTA-free protease inhibitor cocktail (Roche cOmplete™)) and lysed using a microfluidizer processor (Microfluidics™).

The soluble fraction was isolated by centrifugation at 20,000 × g for 30 min at 4 °C. The extract was loaded onto a 5 mL GSTrap FF column (Cytiva Life Sciences) equilibrated with buffer A. TbEIF4E6 was eluted with 25 mL of buffer B (50 mM Tris–HCl, 10 mM GSH, 5 mM DTT, pH 9.0). Fractions containing the target protein were pooled, dialyzed against 50 mM Tris–HCl, 150 mM NaCl, 0.5 mM EDTA, and 2 mM DTT, pH 8.0, and submitted to digestion with TEV protease (proportion 1:80 w/w) for 3 h at 25 °C. The sample was submitted to centrifugation at 20,000 × g for 10 min at 4 °C to eliminate precipitates and loaded a second time onto a 5 mL GSTrap FF column equilibrated with buffer A to separate the GST tag. The flowthrough and wash fractions containing the target protein were pooled, concentrated to a final volume of approximately 2 mL and loaded onto a Superdex 75 16/600 (Cytiva Life Sciences) equilibrated with 20 mM Tris–HCl, 150 mM NaCl, and 2 mM DTT, pH 8.0.

To produce the TbEIF4G5_79-116 peptide, *E. coli* BL21 Star (DE3) cells were transformed with the pET32a-TbEIF4G5_79-116 expression vector and incubated at 37 °C in LB medium containing the selection antibiotic. Expression was induced at 18 °C (OD_600_ ~ 0.7) for sixteen hours with 0.25 mM IPTG. Cells from 2 L culture were harvested by centrifugation, resuspended in 20 mL of buffer A (50 mM Tris–HCl, 200 mM NaCl, 20 mM imidazole, 10 mM β-mercaptoethanol, pH 8.0, supplemented with EDTA-free protease inhibitor cocktail (Roche cOmplete™)) and lysed using a microfluidizer processor (Microfluidics™). The soluble fraction was isolated by centrifugation at 20,000 × g for 30 min at 4 °C. The extract was loaded onto a 5 mL HisTrap HP column (Cytiva Life Sciences) equilibrated with buffer A. The target protein was purified using a two-step gradient, a first linear gradient from 0 to 10% of buffer B (50 mM Tris–HCl, 200 mM NaCl, 500 mM imidazole, 10 mM β-mercaptoethanol, pH 8.0) in 4 column volumes (CV), followed by a linear gradient from 10 to 100% of buffer B in 7 CV. The fractions containing the target protein were pooled, dialyzed and submitted to cleavage with TEV protease using the protocol described above. The sample was submitted to centrifugation to eliminate precipitates and loaded a second time onto a 5 mL HisTrap HP column equilibrated with buffer A to separate the TRX tag. The flow-through fraction containing the peptide was concentrated to a final volume of approximately 4 mL and loaded onto a Superdex 75 16/600 equilibrated with 20 mM Tris–HCl, 150 mM NaCl, and 2 mM DTT, pH 8.0. The purified TbEIF4G5_79-116 peptide was concentrated to approximately 0.5 mM and stored at − 80 °C.

The TbEIF4E6-4G5 complex was purified after incubation of the TbEIF4E6 sample from the second GST-affinity chromatography (after removal of the GST tag) with the purified 4G5 peptide (molar ratio of 4E6/4G5: 1/1.1). The sample was concentrated to a final volume of approximately 2 mL and loaded onto a Superdex 75 16/600 equilibrated with 20 mM Tris–HCl, 150 mM NaCl, and 2 mM DTT, pH 8.0.

The recombinant TbEIF4E6-H51A mutant and the TbEIF4E6-H51A/TbEIF4G5_79-116 complex were produced and purified following the same procedure used for the wild-type TbEIF4E6 and the TbEIF4E6-4G5 complex.

### Nano differential scanning fluorimetry (nanoDSF)

The cap-4 analogue was obtained by applying a liquid-phase oligonucleotide synthesis strategy as previously described^31^. The purified TbEIF4E6, TbEIF4E6-H51A and respective TbEIF4E6-4G5 complexes in buffer containing 20 mM Tris–HCl, 150 mM NaCl, and 2 mM DTT, pH 8.0, were submitted to thermal denaturation on a Prometheus Panta device (NanoTemper Technologies) in the presence and absence of cap-4 (molar ratio of protein/ligand: 1/1.5). For nanoDSF analysis, the samples were submitted to a linear thermal ramp from 20 to 80 °C. Measurements were performed in triplicate using three independent capillaries per sample. Data were analyzed using the PR. Panta Analysis Software (NanoTemper Technologies).

### Crystallization, data collection and processing

The homogeneity of the protein samples was analyzed by DLS and nanoDSF prior to crystallization using a Prometheus Panta device (NanoTemper Technologies). Only monodispersed samples were used in the crystallization tests. TbEIF4E6 and TbEIF4E6-4G5 complexes, prior to and after incubation with cap-4 (molar ratio of protein/ligand: 1/1.5), were submitted to crystallization trials by the sitting drop vapour-diffusion method using commercial screens on a Mosquito® pipettor device (SPT Labtech). Promising conditions were obtained for the TbEIF4E6-4G5 complex and for TbEIF4E6 only in the presence of the cap-4 analogue. Optimization of the initial crystallization conditions was performed by varying the precipitant and protein concentrations. Suitable crystals for diffraction experiments were obtained for the TbEIF4E6-4G5 complex. The best crystals were obtained by hanging drop vapour diffusion (total drop volume of 2 µL, manually prepared) at 18 °C in conditions derived from the Morpheus® Screen (Molecular Dimensions, Sheffield, United Kingdom). TbEIF4E6-4G5 crystals were obtained by mixing the complex at 12 mg/mL in 20 mM Tris–HCl, 150 mM NaCl, 2 mM DTT, pH 8.0 (preincubated with cap-4) with crystallization buffer containing 33% Precipitant Mix 4 (11% v/v MPD; 11% w/v PEG 1000; 11% w/v PEG 3350), 0.09 M NPS (0.03 M sodium nitrate, 0.03 M sodium phosphate dibasic, 0.03 M ammonium sulfate), 0.12 M Ethylene glycols (0.03 M diethylene glycol; 0.03 M triethylene glycol; 0.03 M tetraethylene glycol; 0.03 M pentaethylene glycol), and 0.1 M Buffer System 3 (pH 8.5) (0.05 M Tris base; 0.05 M bicine). TbEIF4E6 crystals were obtained by mixing the protein at 25 mg/mL in 20 mM Tris–HCl, 150 mM NaCl, and 2 mM DTT, pH 8.0 (preincubated with cap-4), with crystallization buffer containing 28.5% Precipitant Mix 1 (19% v/v PEG 500 MME; 9.5% w/v PEG 20000), 0.057 M Divalents (0.0285 M magnesium chloride hexahydrate; 0.0285 M calcium chloride dihydrate), and 0.095 M Buffer system 1 (pH 6.5) (0.057 M MES; 0.038 M imidazole).

X-ray diffraction data were collected at the PROXIMA 2A beam line of the Synchrotron SOLEIL using an EIGER 16M detector (Dectris) and at the MANACÁ beam line of the Synchrotron SIRIUS using a PILATUS 2M detector (Dectris). Diffraction data were processed with the XDS package^50^, and anisotropic correction was performed using STARANISO^51^. Data statistics are presented in Table 1.

### Structure determination, refinement, and analysis

The structure of TbEIF4E6-4G5 was determined by molecular replacement using MOLREP^52^. A TbEIF4E6 model predicted by AlphaFold^34^ was used as the search model. Refinement of the structure was performed by alternating cycles of BUSTER^53^ with visual inspection and rebuilding using COOT^54^. Model validation was performed using MolProbity^55^. Electrostatic potential calculations were performed using Adaptive Poisson-Boltzmann Solver (APBS) software^56^ through the software Chimera^57^. The atomic coordinates and structure factors have been deposited in the Protein Data Bank (code 8UH1).

### Supplementary Information

Supplementary Information.

## Data Availability

The atomic coordinates and structure factors have been deposited in the Protein Data Bank (code 8UH1). The data supporting the findings of this study are available from the corresponding authors upon request.
