# Supplementary material for: Structural analysis of the Trypanosoma brucei EIF4E6/EIF4G5 complex reveals details of the interaction between unusual eIF4F subunits
Source: Sci Rep. 2024 Jan 25;14:2178. doi: 10.1038/s41598-024-52364-1 (PMC10810786; doi:10.1038/s41598-024-52364-1)
Supplement: Supplementary file 1 — Supplementary Information. [file 41598_2024_52364_MOESM1_ESM.pdf]

Supplementary Data

Structural analysis of the *Trypanosoma brucei* EIF4E6/EIF4G5 complex reveals details of the interaction between unusual eIF4F subunits

Renato Ferras Penteado, Renata Santana da Silva, Danielle Maria Nascimento Moura, Gustavo Barbosa de Lima, Amaranta Muniz Malvezzi, Tallyta Tâmara da Silva Monteiro, Camila Cavalcanti Xavier, Sophie Vichier-Guerre, Laurence Dugué, Sylvie Pochet, Nilson Ivo Tonin Zanchin, Christian Robson de Souza Reis, Osvaldo Pompílio de Melo Neto and Beatriz Gomes Guimarães

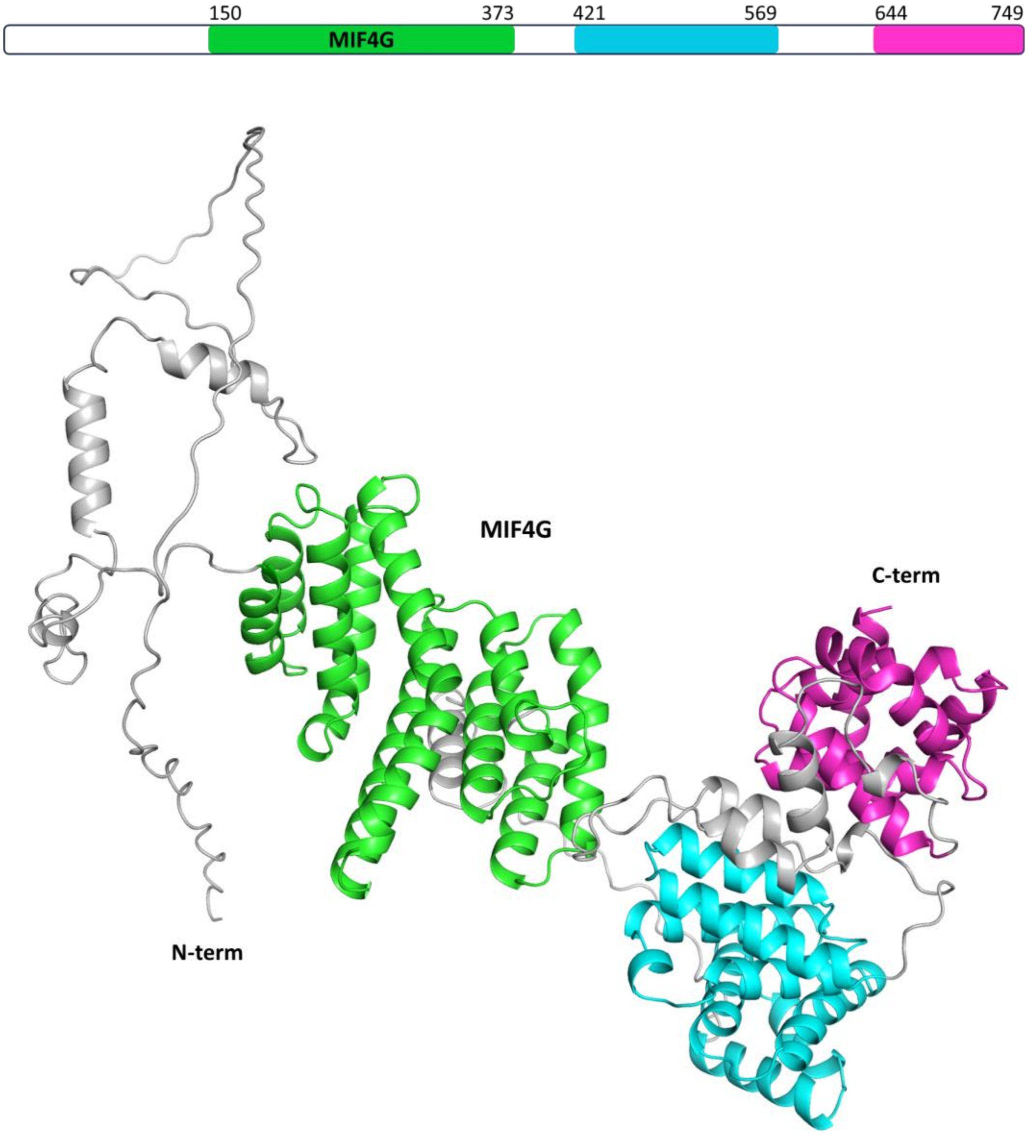

Figure S1: Three-dimensional structure prediction of *T. brucei* EIF4G5. The atomic coordinates were obtained from the AlphaFold Protein Structure Database (https://alphafold.ebi.ac.uk/entry/Q57VY5). The regions with per-residue confidence score (pLDDT) lower than 70, indicating low model confidence, are colored in gray. Three alpha-helical domains, including the MIF4G domain, were modelled with high confidence. The schema on the top indicates the residue numbers corresponding to the boundaries of the globular domains.

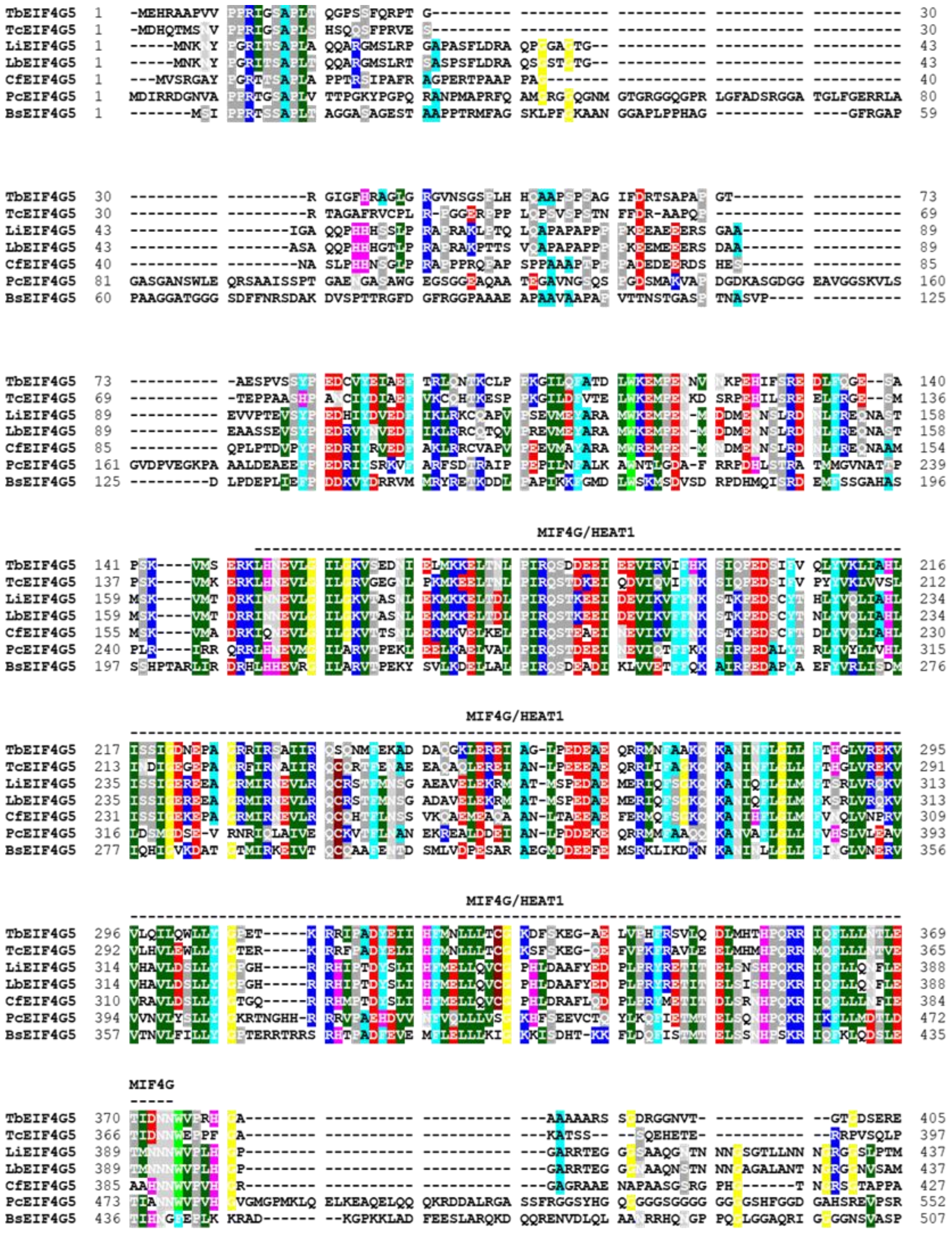

TbEIF4G5
TcEIF4G5
LiEIF4G5
LbEIF4G5
CfEIF4G5
PcEIF4G5
BsEIF4G5
MIF4G/HEAT1
MIF4G

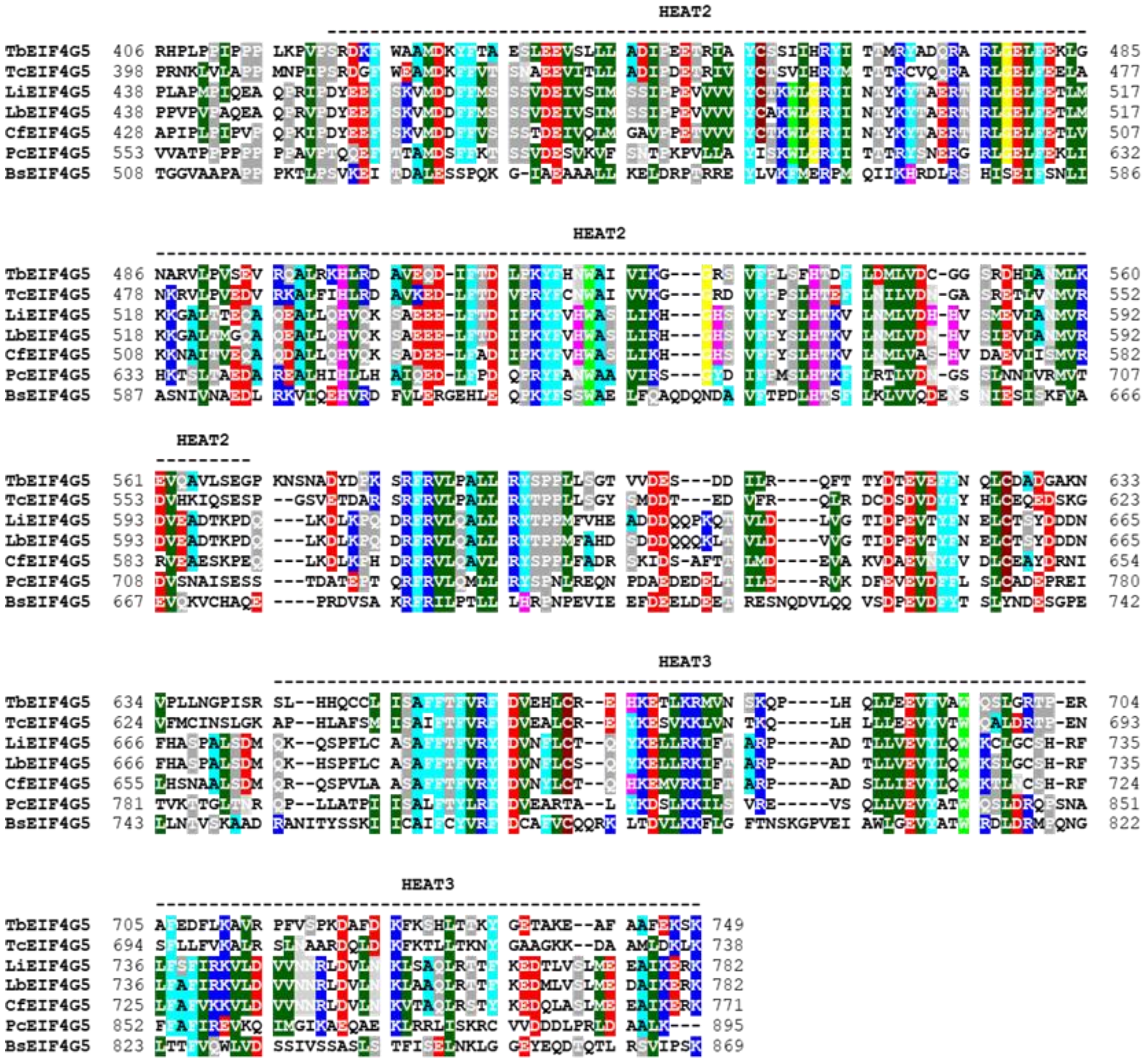

Figure S2: Sequence alignment comparing various kinetoplastid EIF4G5 orthologues. *Trypanosoma brucei* (Tb), *T. cruzi* (Tc), *Leishmania infantum* (Li), *L. braziliensis* (Lb), *Crithidia fasciculata* (Cf), *Paratrypanosoma confusum* (Pc) and Bodo saltans (Bs). The three predicted helical domains (MIF4G/HEAT1, HEAT2 and HEAT3) are indicated. The alignment was carried out using the ClustalW Multiple alignment tool available within the BioEdit Sequence Alignment Editor software (version 7.0.5.3) and the ClustalW2 website (http://www.ebi.ac.uk/Tools/msa/clustalw2/) MAFFT. Occasional manual refinement of the alignment was performed.

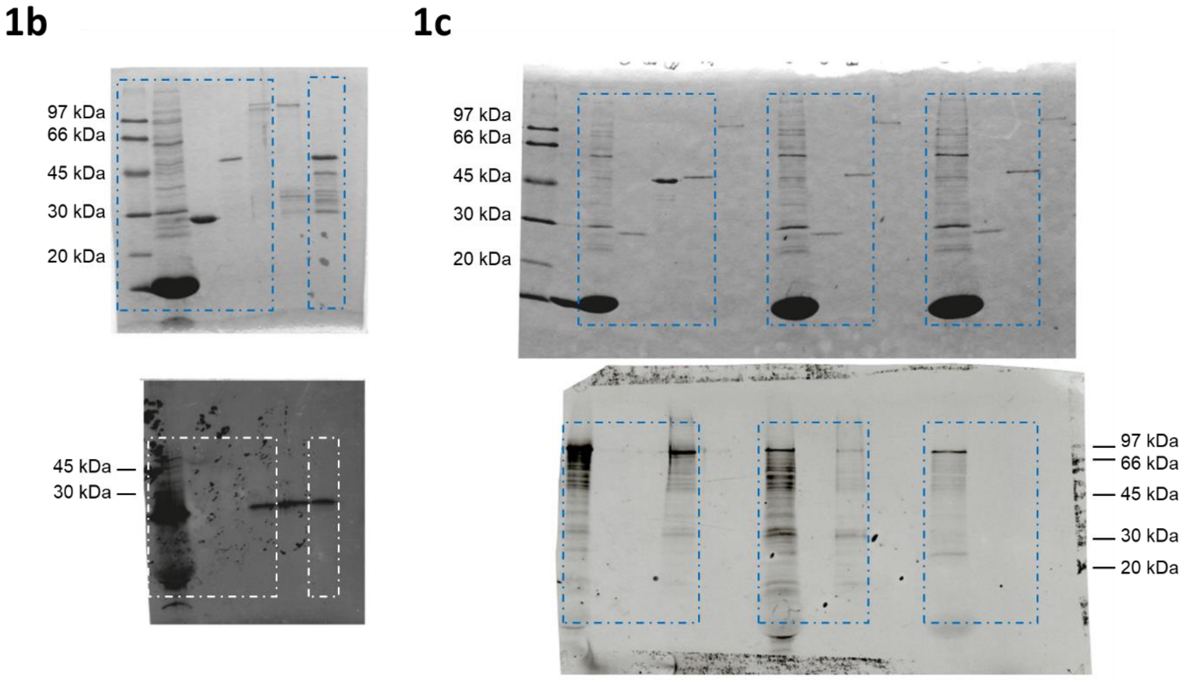

Figure S3: Complete images of the SDS-PAGEs and autoradiographs shown in figures 1b and 1c. The SDS-PAGEs stained with Coomassie blue are shown on the top and the autoradiographs are shown on the bottom. The regions used in the figures of the main text are indicated by dashed boxes.

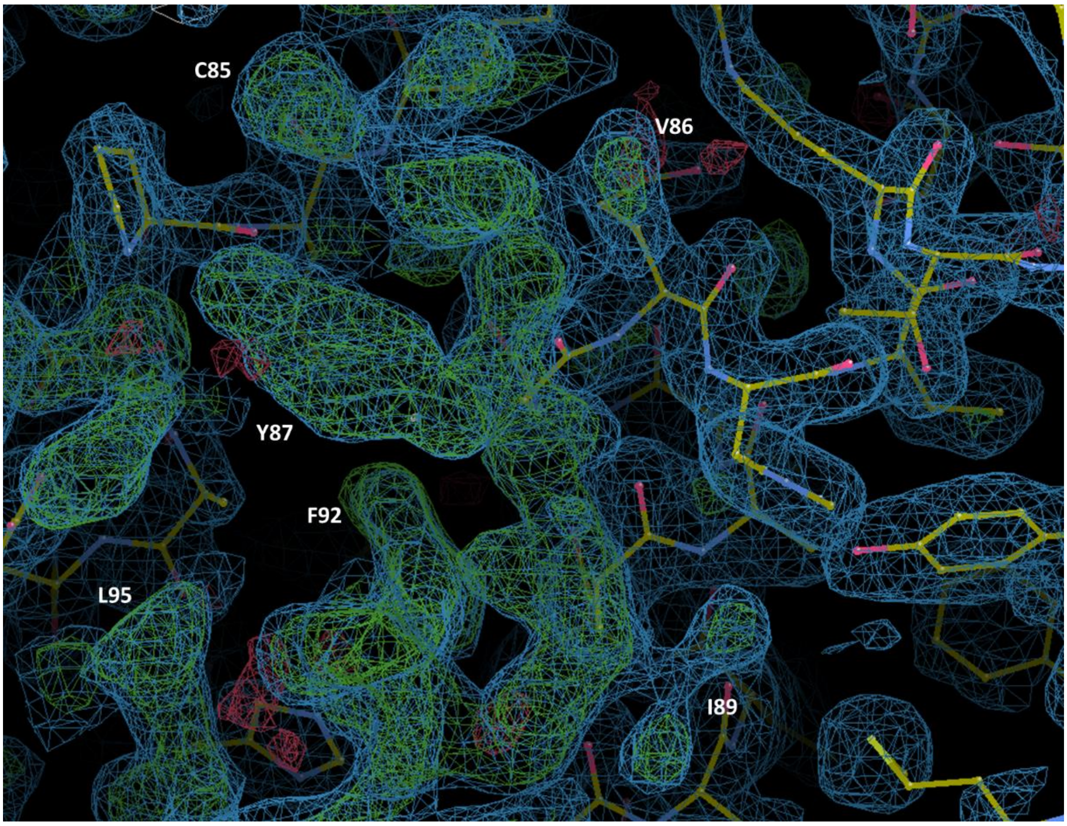

Figure S4: Electron density maps showing a region in the TbEIF4E6-4G5 interface. The 2mFo-DFc electron density map is represented in blue and contoured at 1.0 sigma whereas the mFo-DFc map is represented in green and contoured at 3.0 sigma. The maps were generated after the first refinement cycle of the TbEIF4E6 model (yellow sticks), prior to the building of the 4G5 peptide model. Residues of 4G5 are labelled in white to illustrate the quality of the map.

a

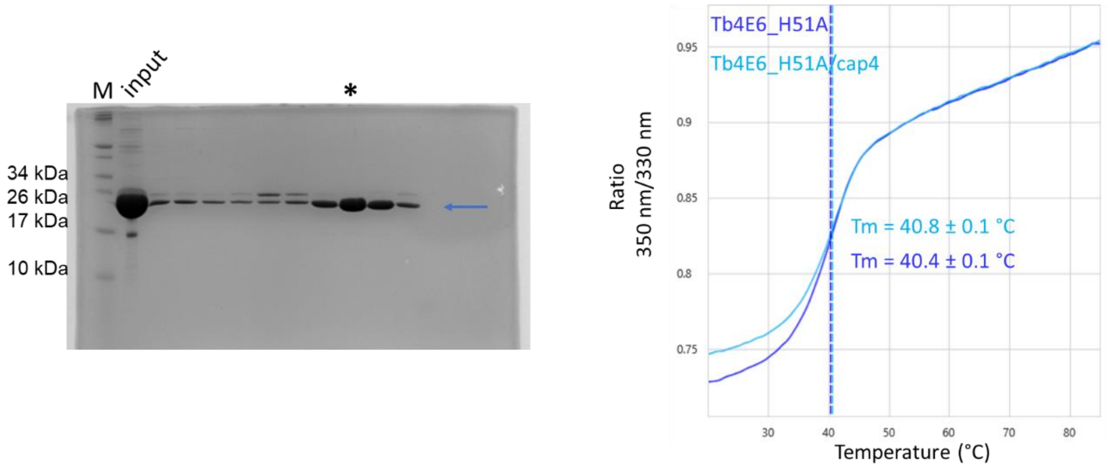

b

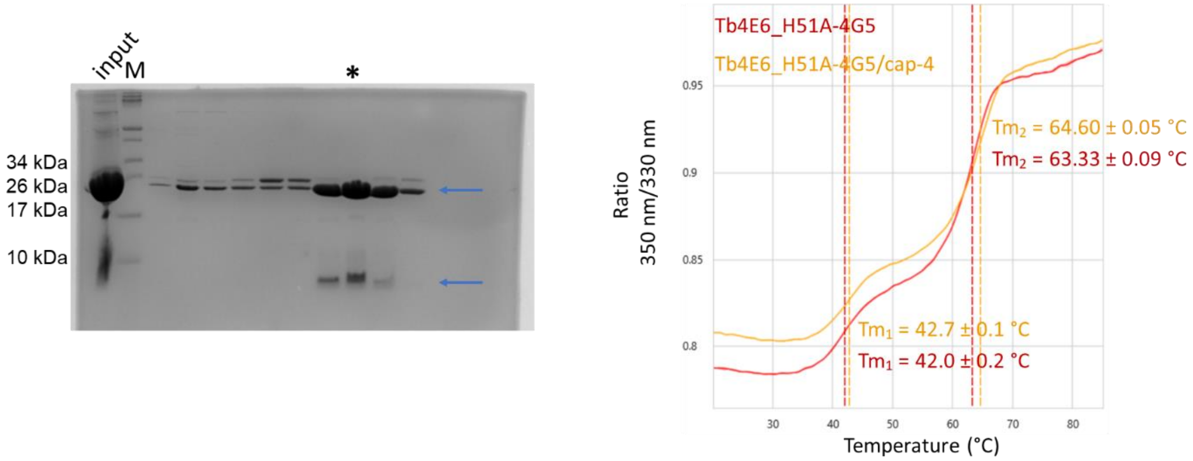

Figure S5: Purification and thermal stability analysis of the TbEIF4E6-H51A mutant protein (a) and of its complex with the TbEIF4G5_79-116 peptide (b). Left panels show SDS-PAGE analysis of the last purification step of recombinant *T. brucei* EIF4E6-H51A mutant (a), and of the TbEIF4E6-H51A/TbEIF4G5_79-116 complex (b). The arrows indicate the relevant bands. The fractions indicated by an asterisk were used for stability analysis. Right panels show the thermal unfolding analyses performed by nanoDSF, in the presence and absence of cap-4. The ratio of emission intensity at 350 nm and 330 nm wavelengths is plotted as a function of temperature and the transition temperature (Tm) is determined by the inflection point. Tm values are indicated in the figure. The TbEIF4E6-H51A/TbEIF4G5_79-116 complex (b) present two transition temperatures possibly corresponding to the monomeric TbEIF4E6-H51A (Tm_1) and to the complex (Tm_2). Sample incubation with cap-4 analogue does not significantly change any of the transition temperatures.

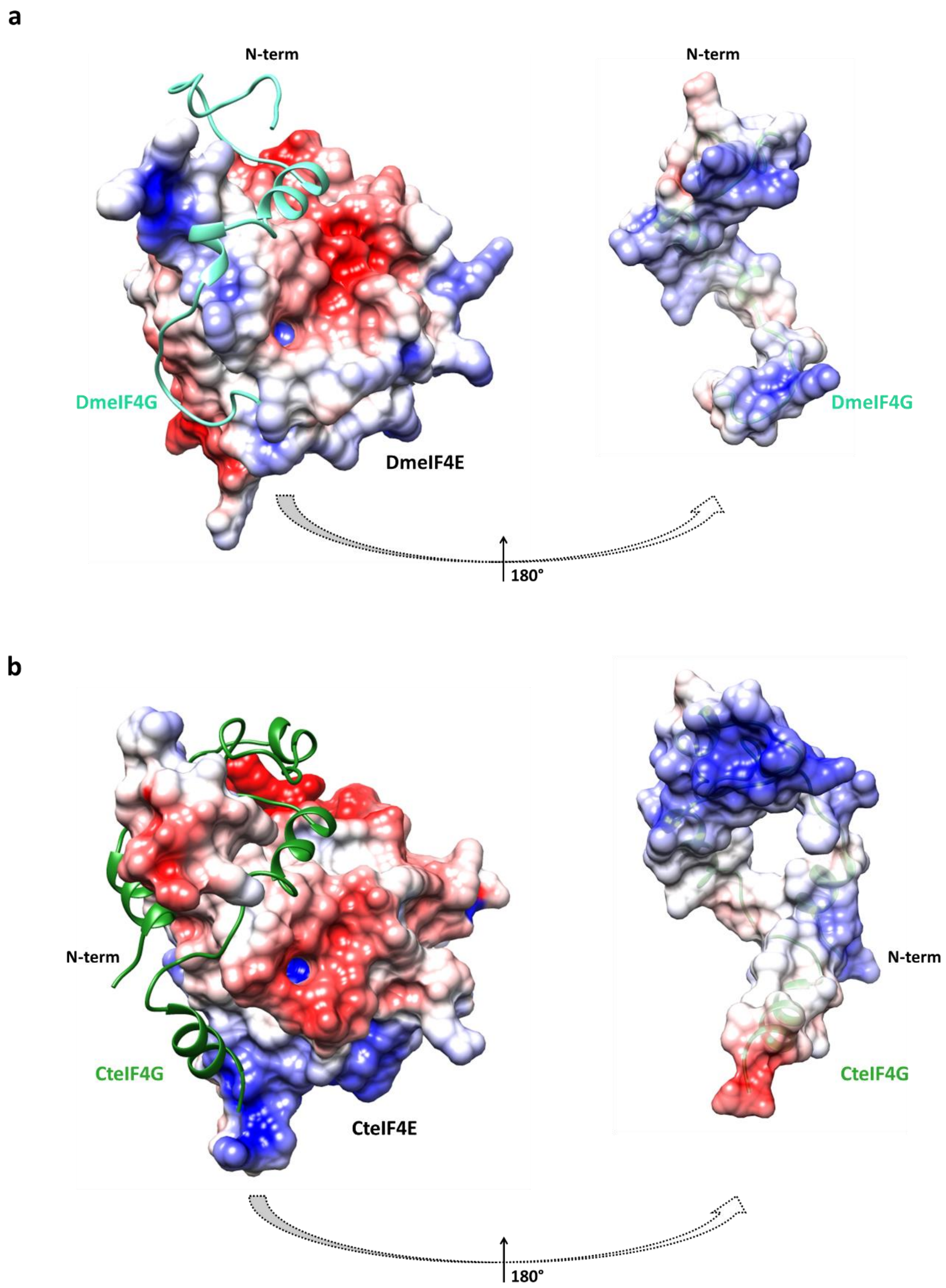

Figure S6: Electrostatic potential surfaces of *Drosophila melanogaster* eIF4E-4G (a) and *Chaetomium thermophilum* eIF4E-4G (b) complexes. The analysis was carried out using the atomic coordinates available at the Protein Data Bank under codes 5T47 (DmeIF4E-4G) and 6FC0 (CteIF4E-4G). The left panels show the electrostatic potential surfaces of eIF4E with the 4G fragments represented in ribbons. The right panels show the electrostatic potential surfaces of the 4G peptides. The 4G peptide models are turned by 180 degrees relative to the orientation shown on the left to display their interaction surface. The boundaries for potential contour map visualization are - 5 kT/e (red) and +5 kT/e (blue).

Table S1: Sequence of the synthetic oligonucleotides used for site-directed mutagenesis of TbEIF4E6 and TbEIFG5 and generation of the pGEX4T3TEV2 vector. The nucleotides mutated relative to the *Trypanosoma brucei brucei* TREU927 reference genome sequence are indicated in blue and bold. The sequence encoding the new *Nde*I site in the pGEX4T3TEV2 vector is in red and bold with the sequence encoding the TEV site (ENLYFQG) underlined.

**Gene/ mutation**	Primer sequence 5' – 3'
EIF4E6 H51A_F	AAAAAGCATTTATTGTAGCG**GC**CAGCTCCTCTGCTGTCGAG
EIF4E6 H51A_R	CTCGACAGCAGAGGAGCTG**GC**CGCTACAATAAATGCTTTTT
EIF4G5 EF_F	TTGTGTTCTGCAATCTGGTA**GC**C**G**CAGCGATCTCGTAGACGCAG
EIF4G5 EF_R	CTGCGTCTACGAGATCGCTG**C**G**GC**TACCAGATTGCAGAACACAA
EIF4G5 VY_F	GCTACCCGGAAGACTGGCG**CC**G**GC**CGAGATCGCTGAGTTTAC
EIF4G5 VY_R	GTAAACTCAGCGATCTCG**GC**G**G**CGCAGTCTTCCGGGTAGC
pGEX4T3 TEV_F	GATCT**CATATG**GAAAATCTGTACTTCCAAGGCG
pGEX4T3 TEV_R	GATCCGCCTTGGAAGTACAGATTTTC**CATATG**A
